# Publisher Correction: Re-evaluation of battery-grade lithium purity toward sustainable batteries

**DOI:** 10.1038/s41467-024-46157-3

**Published:** 2024-02-26

**Authors:** Gogwon Choe, Hyungsub Kim, Jaesub Kwon, Woochul Jung, Kyu-Young Park, Yong-Tae Kim

**Affiliations:** 1 Department of Materials Science and Engineering, Pohang University of Science and Technology, 77 Cheongam-Ro, Nam-Gu, Pohang, Gyeongbuk 37673 Republic of Korea; 2 Neutron Science Division, Korea Atomic Energy Research Institute (KAERI), 111 Daedeok-daero 989 Beon-Gil, Yuseong-gu, Daejeon 34057 Republic of Korea; 3 Lithium Materials Research Group, Research Institute of Industrial Science and Technology (RIST), 67 Cheongam-Ro, Nam-Gu, Pohang, Gyeongbuk 37673 Republic of Korea; 4 Graduate Institute of Ferrous & Energy Materials Technology, Pohang University of Science and Technology, 77 Cheongam-Ro, Nam-Gu, Pohang, Gyeongbuk 37673 Republic of Korea

**Keywords:** Batteries, Chemical engineering, Batteries

Correction to: *Nature Communications* 10.1038/s41467-024-44812-3, published online 08 February 2024

The original version of the Peer Review File associated with this Article was updated shortly after publication to redact unpublished data in Page 11 and 19.

### Supplementary information

Updated Peer Review File

