# Supplementary material for: Re-evaluation of battery-grade lithium purity toward sustainable batteries
Source: Nat Commun. 2024 Feb 8;15:1185. doi: 10.1038/s41467-024-44812-3 (PMC10853534; doi:10.1038/s41467-024-44812-3)
Supplement: Supplementary file 1 — Supplementary Information [file 41467_2024_44812_MOESM1_ESM.pdf]

Supplementary Information

Re-evaluation of Battery-grade Lithium Purity Toward Sustainable Batteries

Gogwon Choe[a], Hyungsub Kim[b], Jaesub Kwon[a], Woochul Jung*[c], Kyu-Young Park*[a,d], and Yong-Tae Kim*[a,d]

[a]Department of Materials Science and Engineering, Pohang University of Science and Technology; 77 Cheongam-Ro, Nam-Gu, Pohang, Gyeongbuk 37673, Republic of Korea

[b]Neutron Science Division, Korea Atomic Energy Research Institute (KAERI); 111 Daedeok-daero 989 Beon-Gil, Yuseong-gu, Daejeon, 34057, Republic of Korea

[c]Lithium Materials Research Group, Research Institute of Industrial Science and Technology (RIST); 67 Cheongam-Ro, Nam-Gu, Pohang, Gyeongbuk 37673, Republic of Korea

[d]Graduate Institute of Ferrous & Energy Materials Technology, Pohang University of Science and Technology; 77 Cheongam-Ro, Nam-Gu, Pohang, Gyeongbuk 37673, Republic of Korea

*Corresponding author. Email: yongtae@postech.ac.kr; kypark0922@postech.ac.kr; wcjung@rist.re.kr

Inventory of Supporting Information

- Supplementary Figures S1 to S38
- Supplementary Note S1 to S5
- Supplementary Tables S1 to S23
- Supplementary Texts

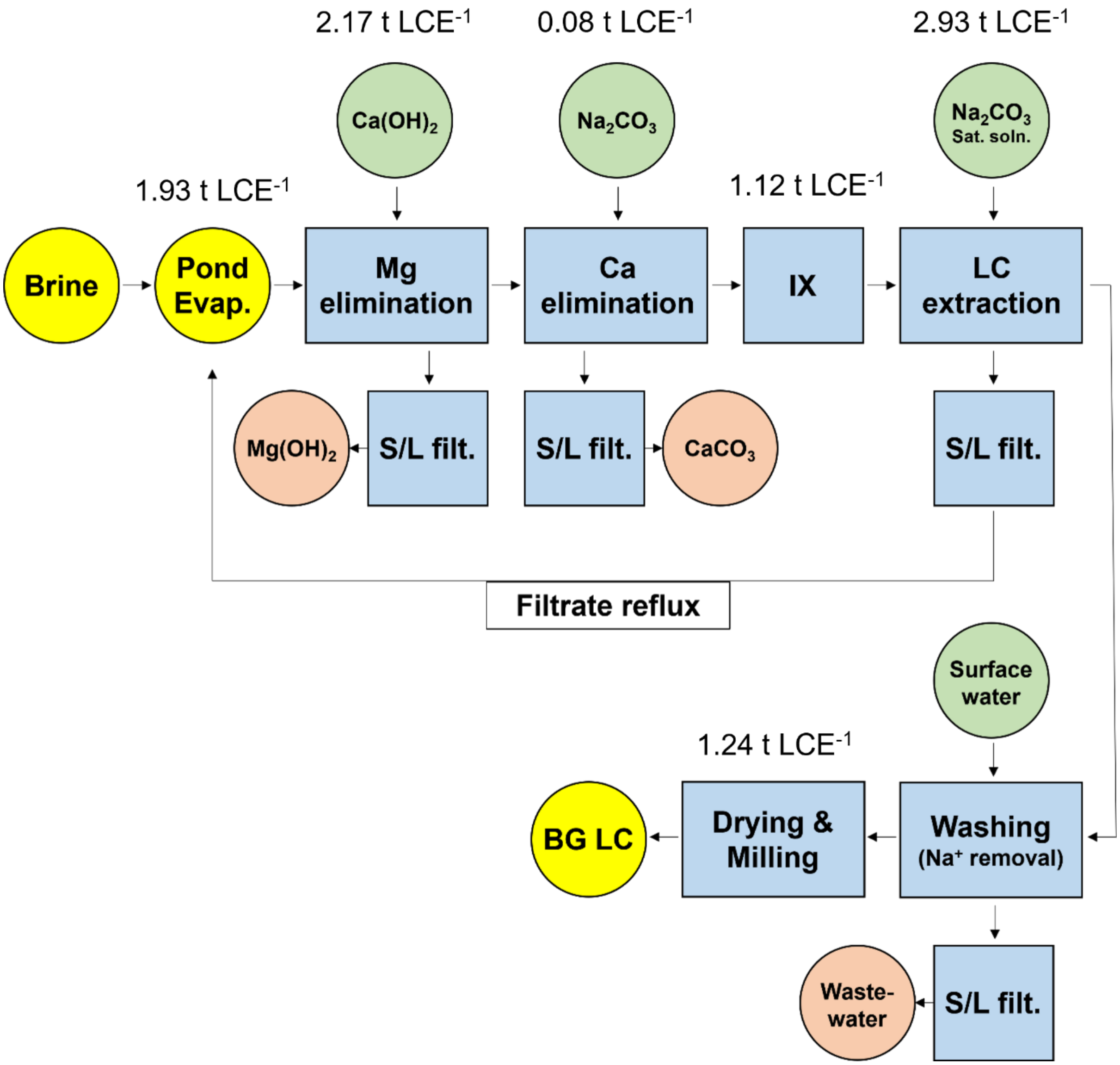

Supplementary Fig. S1. Process diagram of commercial LC extraction process from brine. The CO_2 footprints of each step are indicated above the diagrams.

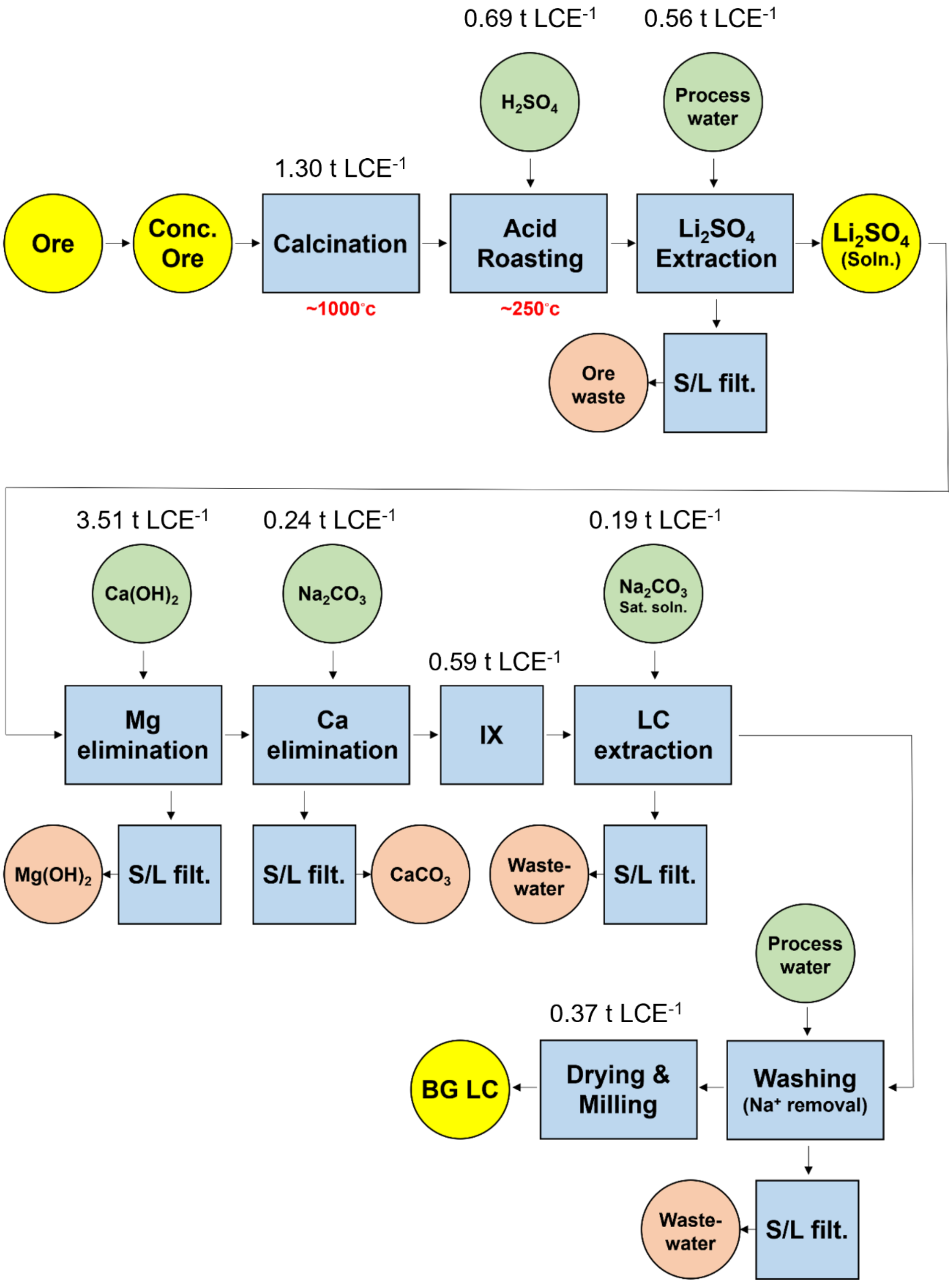

Supplementary Fig. S2. Process diagram of commercial LC extraction process from hard rock. The CO_2 footprints of each step are indicated above the diagrams.

Supplementary Text for Supplementary Figs. S1 and S2

Brine) The Li brine contains ~1.0 g L^{-1} which is low concentration for purifying process. Thus, the concentration of lithium to ~4.0 g L^{-1} is firstly adjusted through natural evaporation in ponds.

Hard rock) The ore is concentrated by grinding and separation methods. Next, the concentrated ore go through calcination process transforming phase applicable to leaching process (acid roasting); α-spodumene to β-spodumene. As temperature increases, coarse α-spodumene turn into porous β-spodumene by thermal expansion. Following acid roasting process leaches minerals from ore to produce Li_2SO_4 solution for the next purifying process.

Purifying process) Representative impurity elements of Mg and Ca are eliminated with $Mg(OH)_2$ and $CaCO_3$ form by using $Ca(OH)_2$ and Na_2CO_3 reagents, respectively. The further concentration between Mg elimination and Ca elimination is optional.

$$Ca(OH)_2\,(s) + \,Mg^{2+}\,(aq) \rightarrow Ca^{2+}\,(aq) + Mg(OH)_2\,(s) \tag{1}$$

$$Na_2CO_3\,(s) + \,Ca^{2+}\,(aq) \rightarrow 2Na^{+}\,(aq) + CaCO_3\,(s) \tag{2}$$

Then, ion exchange (IX) process further increases purity to meet the *battery-grade* over 99.5%. Now, the materials are ready for LC extraction process. Na_2CO_3 saturated solution is put into LC extraction batch to precipitate Li_2CO_3. Produced Li_2CO_3 is washed to eliminated Na ion and dried/milled to produce *battery-grade* Li_2CO_3.

Supplementary Table S1. CAPEX (capital expenditure) breakdown of commercial brine process and approximated separation of refining process cost. Operating cost is generally proportional to CAPEX that refining process cost ratio of OPEX (operating expenditure) is analogous to CAPEX refining process cost ratio. Because of the ambiguity in separation of refining process cost of OPEX (due to inner feedback/bypass or reflux in operation), estimated refining process cost ratio of 29% from total production cost is given here.

Brine Process

CAPEX*		Cost (M$)	Refining process cost (M$)
Brine area	Pond Civil.	145.7	-[1]
	Equipment cost	27.4	
	Installation cost	48.9	
	Indirect cost	64.2	
	Brine area total	286.2	-
Refinery factory	Equipment cost	34.4	118.4[2]
	Installation cost	54.7	
	Indirect cost	29.3	
	Refinery factory total	118.4	118.4
Total		404.6	118.4[3]

*Process capacity Li_2CO_3 25,000 ton/year

[1]Brine pump and pond evaporation, Li_2CO_3 crude production

[2]Total refinery factory cost for refining process cost

[3]29% of CAPEX for refining process

Supplementary Table S2. CAPEX (capital expenditure) breakdown of commercial hard rock process and approximated separation of refining process cost. Operating cost is generally proportional to CAPEX that refining process cost ratio of OPEX (operating expenditure) is analogous to CAPEX refining process cost ratio. Because of the ambiguity in separation of refining process cost of OPEX (due to inner feedback/bypass or reflux in operation), estimated refining process cost ratio of 37% from total production cost is given here.

Hard Rock Process

CAPEX*		Cost (M$)	Refining process cost (M$)
Equipment cost	Pyro area	46.2	-
	Hydro area	76.9	46.14[1]
	Reagents & Utility	10.5	3.78[2]
Total equipment cost		133.6	49.92
Bulk and installation cost	Civil	13.4	55.332[3]
	Concrete	20	
	Structural steel	20	
	Mechanical installation	13.4	
	Piping	33.4	
	Electrical & instrumentation	53.5	
Total bulk and installation cost		153.7	55.332
Total		287.3	105.252[4]

*Process capacity Li_2CO_3 25,000 ton/year

[1]60% of hydro area for refining process (rest of 40% for Li_2CO_3 reaction and S/L filtration)

[2]Reagents & utility cost – [pyro + (hydro * 0.4)]

[3]Total bulk and installation cost – [pyro + (hydro * 0.4)]

[4]37% of CAPEX for refining process

Supplementary Note S1:

For better understanding, flow chart summaries of all synthesized materials are provided here.

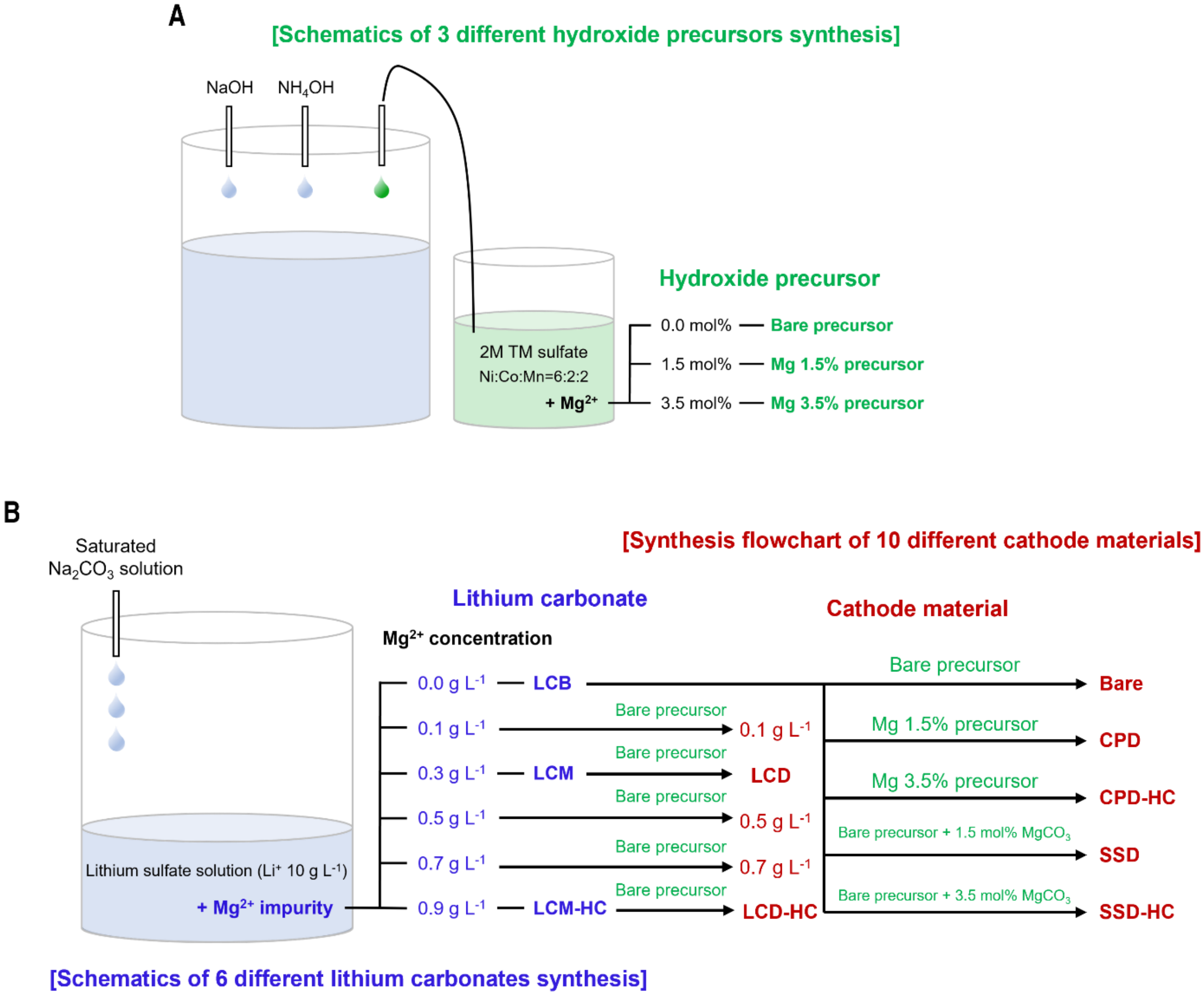

Supplementary Fig. S3. Flow chart summaries of all synthesized materials. (**A**) Schematics of 3 different hydroxide precursors synthesis. (**B**) Schematics of 6 different lithium carbonates synthesis and synthesis flowchart of 10 different cathode materials.

Three transition metal hydroxides of $Ni_{0.6}Co_{0.2}Mn_{0.2}(OH)_2$ with different Mg concentrations were prepared by a typical precipitation method first (**A**). The control $Ni_{0.6}Co_{0.2}Mn_{0.2}(OH)_2$ (Mg

0%) was denoted as a “Bare precursor”. $Ni_{0.6}Co_{0.2}Mn_{0.2}(OH)_2$ with 1.5 mol% and 3.5 mol% of Mg^{2+} were denoted as “Mg 1.5% precursor” and “Mg 3.5% precursor”, respectively.

Next, lithium carbonates with different Mg^{2+} concentrations were prepared by dropping saturated Na_2CO_3 solution into Mg impurity contained lithium sulfate solutions (Li^+ 10g L^{-1} solution) (**B**). Here, six different Mg concentrations in lithium sulfate solutions were prepared with 0.0, 0.1, 0.3, 0.5, 0.7, and 0.9 g L^{-1}. Conditions for 0.0, 0.3, and 0.9 g L^{-1} were denoted as “LCB” (lithium carbonate bare), “LCM” (lithium carbonate magnesium), and “LCM-HC” (lithium carbonate magnesium - high concentration), respectively, for better discussion.

All synthesized lithium carbonates were calcinated with a “Bare precursor” to synthesize $LiNi_{0.6}Co_{0.2}Mn_{0.2}O_2$. In this case, cathode materials made of LCB, LCM, and LCM-HC were specially denoted as “Bare”, “LCD” (lithium carbonate doping), and “LCD-HC” (lithium carbonate doping-high concentration), respectively.

Coprecipitation-doped cathode materials made of Mg 1.5% precursor and Mg 3.5% precursor were denoted as “CPD” (coprecipitation doping) and “CPD-HC” (coprecipitation doping-high concentration), respectively.

Solid-state-doped cathode materials targeting 1.5 mol% and 3.5 mol% of Mg^{2+} doping by adding extra $MgCO_3$ were denoted as “SSD” (solid-state doping) and “SSD-HC” (solid-state doping-high concentration), respectively.

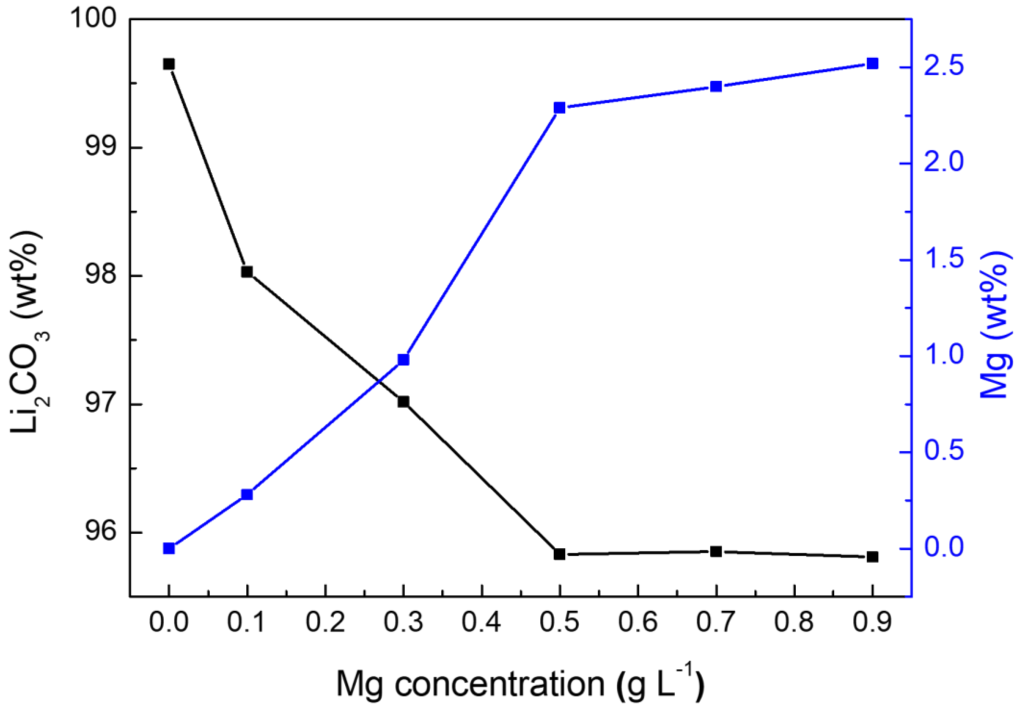

Supplementary Fig. S4. Comparison of purity of Li_2CO_3 and Mg^{2+} impurity content from different Mg^{2+} impurities concentrations. Mg^{2+} concentrations of 0.0, 0.3, and 0.9 g L^{-1} were denoted as LCB, LCM, and LCM-HC, respectively.

Supplementary Table S3. The inductive coupled plasma optical emission spectrometry results of Li_2CO_3 with different Mg^{2+} impurities concentrations. Li^+ and S^{2-} contents were converted to the weights of Li_2CO_3 and sulfate ion. Mg^{2+} concentrations of 0.0, 0.3, and 0.9 g L^{-1} were denoted as LCB, LCM, and LCM-HC, respectively.

Mg concentration (g L^{-1})	Li_2CO_3 (wt%)	Mg (wt%)	SO_4 (wt%)	Na (wt%)
0.0	99.65	N/D	0.31	0.04
0.1	98.03	0.28	1.49	0.19
0.3	97.02	0.98	1.86	0.09
0.5	95.83	2.29	1.51	0.37
0.7	95.85	2.40	1.48	0.28
0.9	95.81	2.52	1.56	0.12

N/D: not detected

Supplementary Text for Supplementary Fig. S4 and Table S3.

The inclusion of the Mg element in Li_2CO_3 increased as Mg^{2+} impurity was added into the Li_2CO_3 reaction batch, while the purity of Li_2CO_3 decreased. In addition, the solubility of Mg^{2+} in Li_2CO_3 is saturated over 0.5 g L^{-1} additive batch, confirming that the limit that Mg impurity can be incorporated in Li_2CO_3 is around 2.5 wt% during the Li extraction process. Here, control LCB satisfies the guideline of *battery-grade* Li-source (> 99.5%), but the others are far from the *battery-grade* guideline.

Supplementary Note S2:

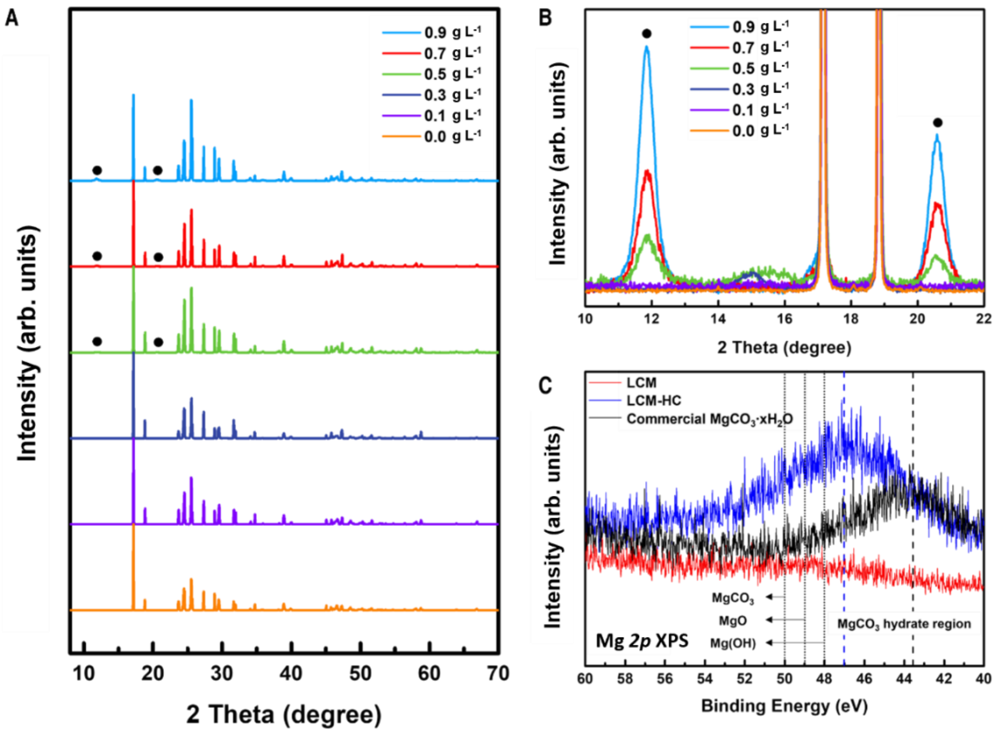

Supplementary Fig. S5. Characterization of synthesized lithium carbonates. XRD patterns of lithium carbonates (**A**) and overlayed enlarged $MgCO_3{\cdot}3H_2O$ peak region (**B**). (**C**) XPS spectrum of LCM, LCM-HC, and commercial $MgCO_3{\cdot}xH_2O$.

XRD analysis of Li_2CO_3 from different Mg^{2+} impurity concentrations were conducted (**A**). The $MgCO_3{\cdot}3H_2O$ phase peaks (JCPDS Card 020-0669) were marked with black circles. Mg^{2+} concentrations of 0.0, 0.3, and 0.9 g L^{-1} were denoted as LCB, LCM, and LCM-HC, respectively. As shown in **Fig. 2a−d**, Mg^{2+} forms solid solution inside the Li_2CO_3, however, over 0.5 g L^{-1} of Mg^{2+} impurity concentration, the phase separation to $MgCO_3{\cdot}3H_2O$ were observed (**B**; overlayed enlarged $MgCO_3{\cdot}3H_2O$ peak region). In addition, this $MgCO_3$ hydrate phases will be more formatted on the surface than inside of Li_2CO_3 particles according to XPS results (**C**). These results make good agreement with ICP-OES data (**Supplementary Fig. S4**) that Mg^{2+} concentration at Li_2CO_3 saturated over 0.5 g L^{-1} followed by the overloaded Mg^{2+} formatting surface $MgCO_3{\cdot}xH_2O$ phases.

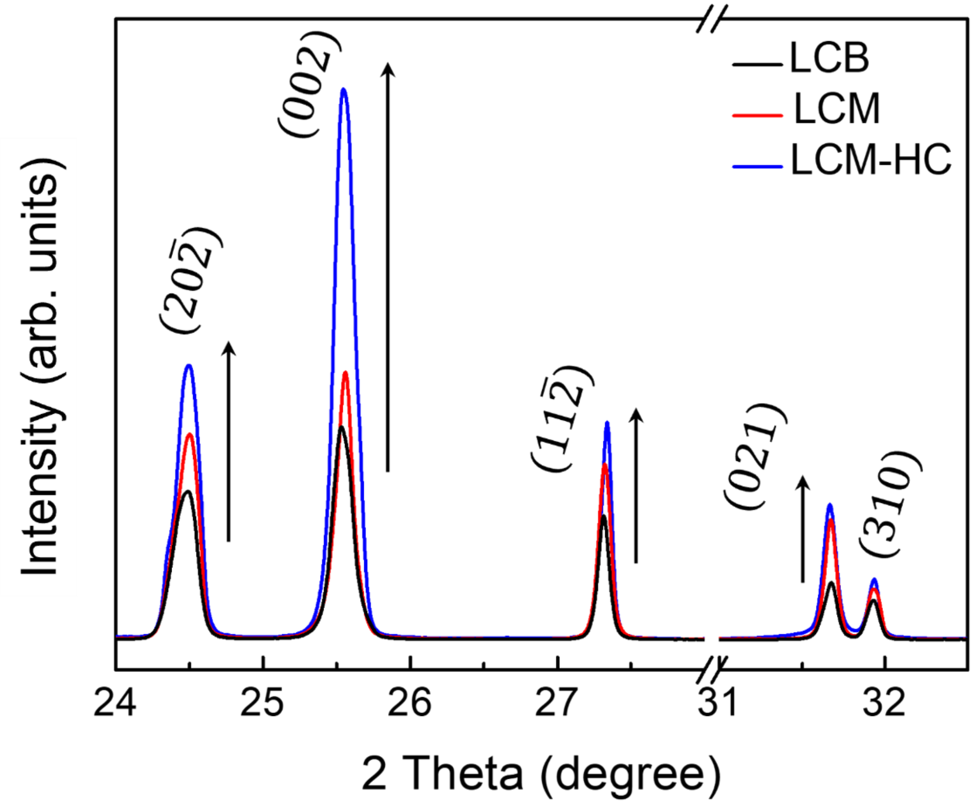

Supplementary Fig. S6. The comparison of XRD patterns of LCB, LCM, and LCM-HC. The obvious increasement of the peak intensity was observed.

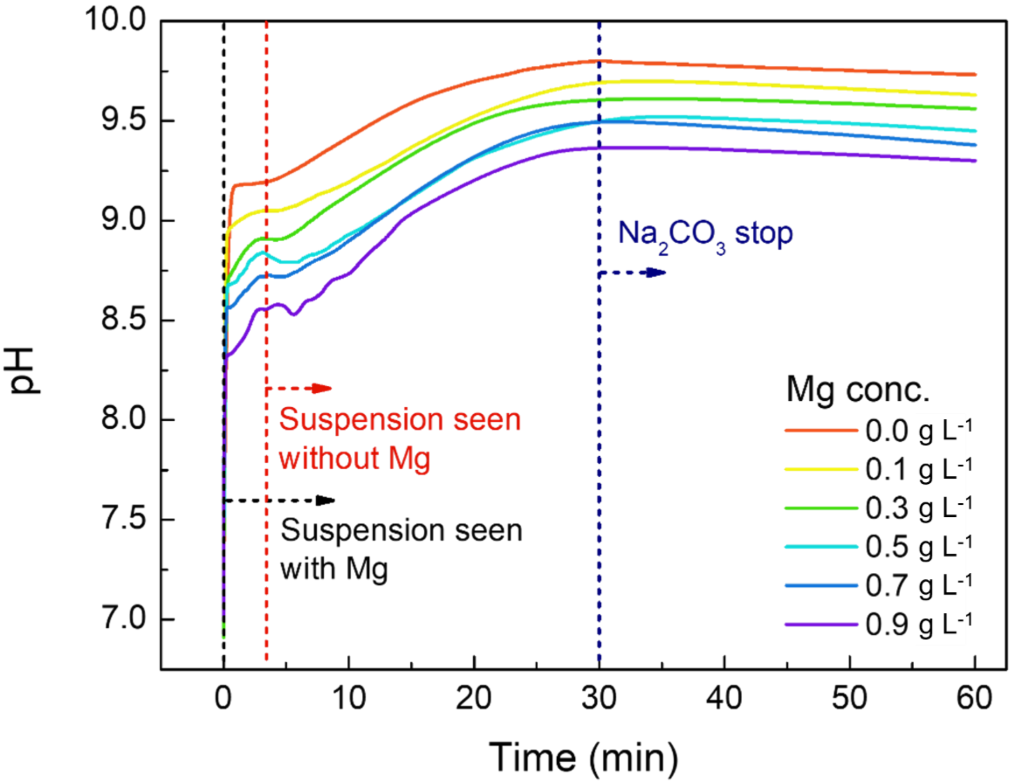

Supplementary Fig. S7. Comparison of pH trends in the Li_2CO_3 precipitation reactor as a function of time with different amounts of Mg^{2+} impurities. The times when white suspension is seen are marked with dashed lines. Suspension was immediately appeared after dropping Na_2CO_3 saturated solution with Mg^{2+} impurity. The time when saturated Na_2CO_3 solution feeding was stopped is marked with dark blue dashed line. Mg^{2+} concentrations of 0.0, 0.3, and 0.9 g L^{-1} were denoted as LCB, LCM-1, and LCM-3, respectively.

Supplementary Text for Supplementary Fig. S7

Corresponding chemical reactions of Li_2CO_3 precipitation[1,2] are below.

$$CO_3{}^{2-}\ (aq) + H_2O\ (l)\ \leftrightarrows\ HCO_3{}^{-}\ (aq) + OH^{-}\ (aq) \tag{3}$$

$$HCO_3{}^{-}\ (aq) + H_2O\ (l)\ \leftrightarrows\ H_2CO_3\ (aq) + OH^{-}\ (aq) \tag{4}$$

$$2Li^{+}\ (aq) + HCO_3{}^{-}\ (aq)\ \longrightarrow\ Li_2CO_3\ (s) + H^{+}\ (aq) \tag{5}$$

CO_3^{2-} ions from Na_2CO_3 saturated solution go through reaction (3) and (4), generating HCO_3^{-}, H_2CO_3, and OH^{-} ions. Generated HCO_3^{-} from reaction (3) reacts with Li^{+} from Li_2SO_4 solution in reaction batch as reaction (5) and deprotonated, followed by generated proton decreasing pH. For those reasons, saturated Na_2CO_3 solution increases pH to ~ 9.2 in reaction batch immediately after pumped into the batch followed by gradual increasement of pH competing with deprotonation reaction as Li_2CO_3 precipitation reaction start. pH gradually decreased as Li_2CO_3 particle grow after Na_2CO_3 saturated solution stopped as shown in pH tracing measurement.

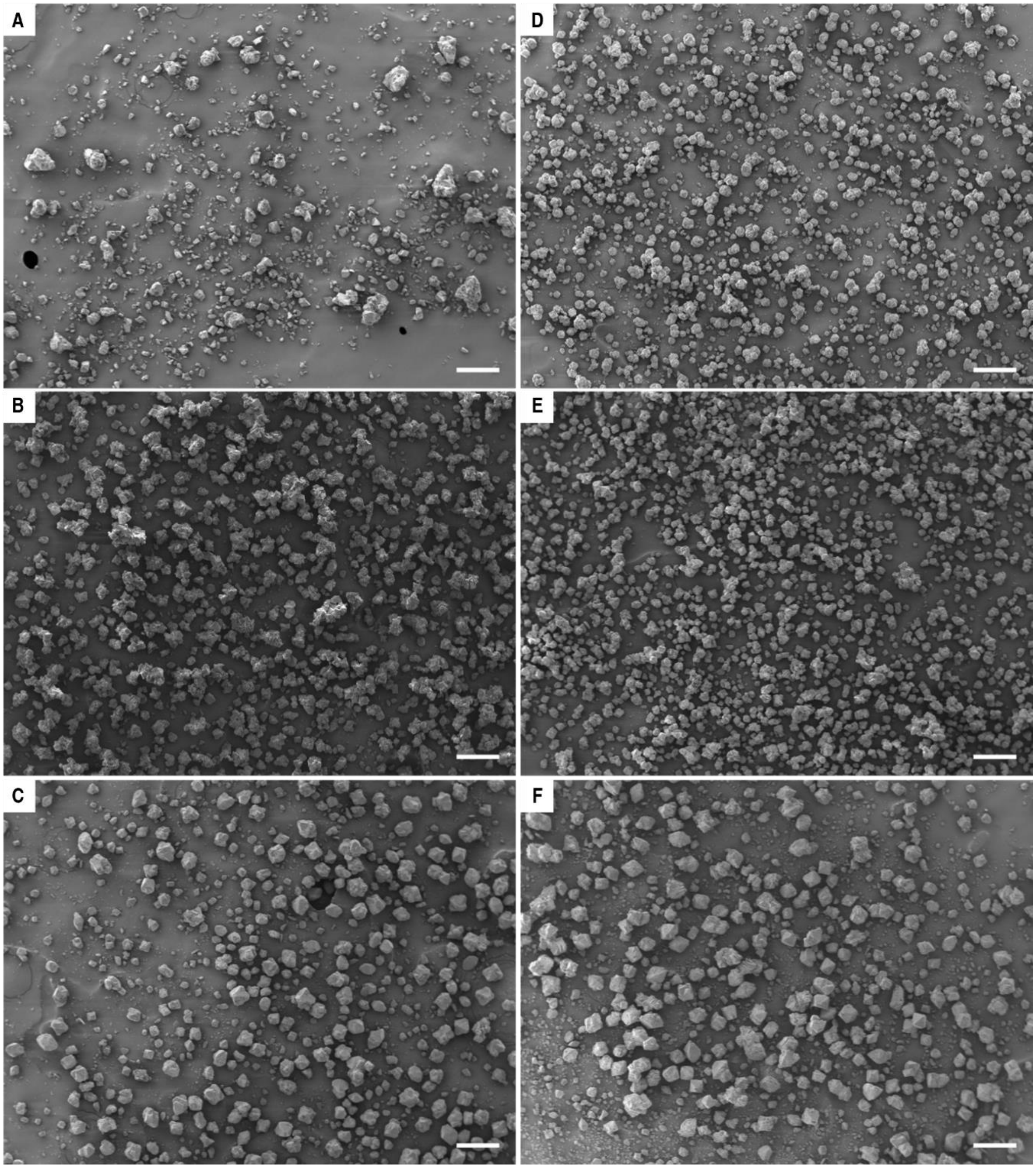

Supplementary Fig. S8. Comparison of particle size distributions and morphologies of Li_2CO_3 with different Mg^{2+} impurity concentration of 0.0 g L^{-1} (**A**), 0.1 g L^{-1} (**B**), 0.3 g L^{-1} (**C**), 0.5 g L^{-1} (**D**), 0.7 g L^{-1} (**E**), and 0.9 g L^{-1} (**F**). Scale bars are corresponding to 100 μm.

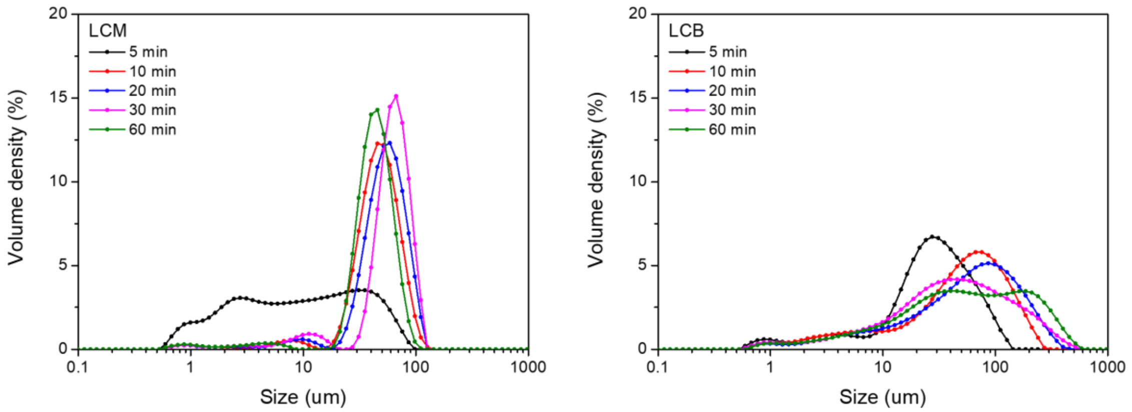

Supplementary Fig. S9. Ex-situ PSD analysis was conducted based on the reaction time of LCM (left) and LCB (right). Smaller-sized particles were more prevalent in the early-stage reaction (~5 min), which is believed to be due to the presence of $MgCO_3 \cdot xH_2O$ nanoparticle species. Subsequently, a narrower PSD was observed at the reaction batch containing Mg impurities.

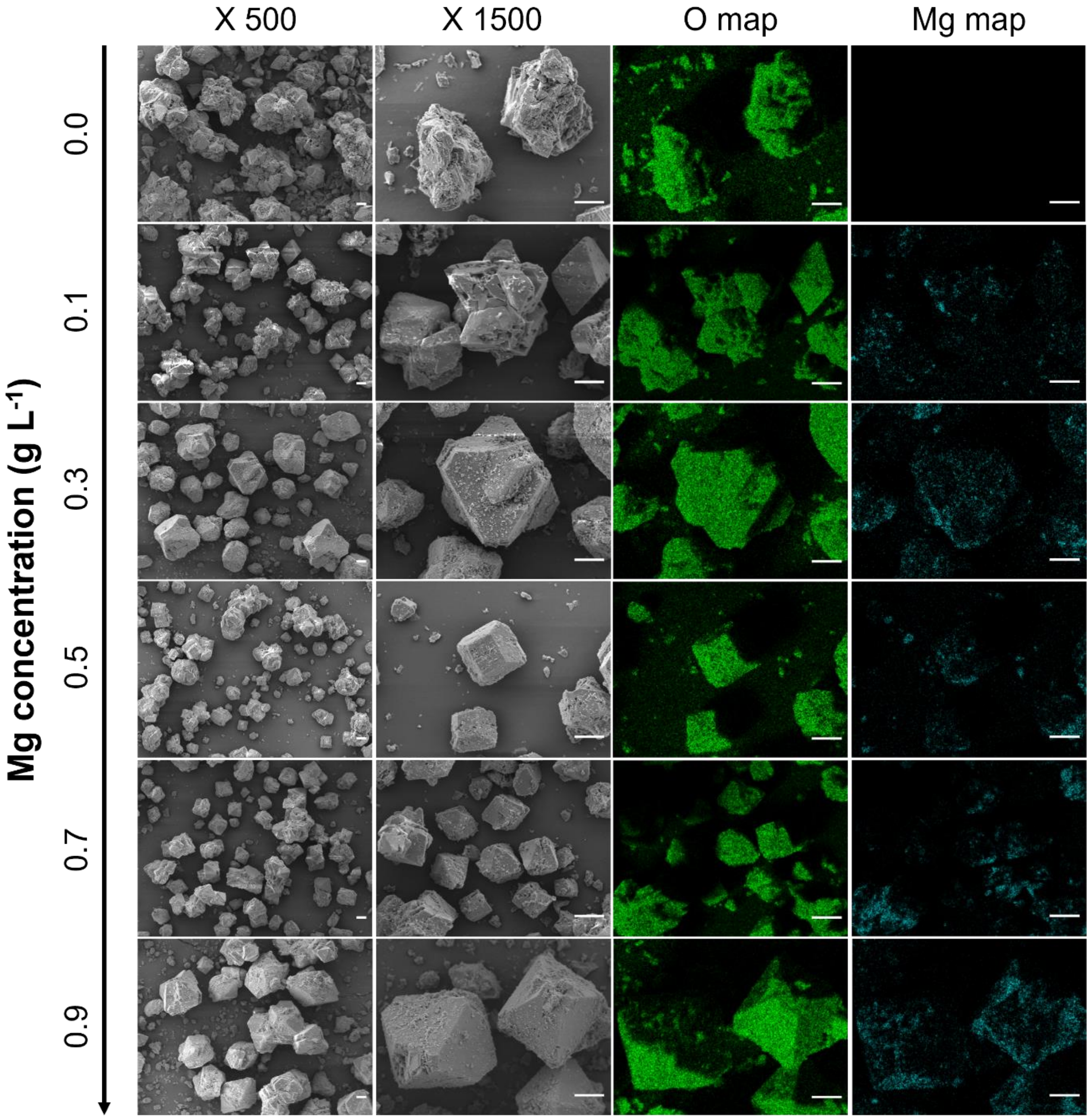

Supplementary Fig. S10. Comparison of SEM-EDS images of Li_2CO_3 synthesized with different Mg^{2+} impurity concentrations. Scale bars, 10 μm. Mg^{2+} concentrations of 0.0, 0.3, and 0.9 g L^{-1} were denoted as LCB, LCM, and LCM-HC, respectively. Further elemental analysis using an energy-dispersive X-ray spectroscopy (EDS) mapping showed an undetectable amount of Mg^{2+} signal at LCB, while homogeneously distributed Mg^{2+} signal was observed in Mg containing Li_2CO_3 particles.

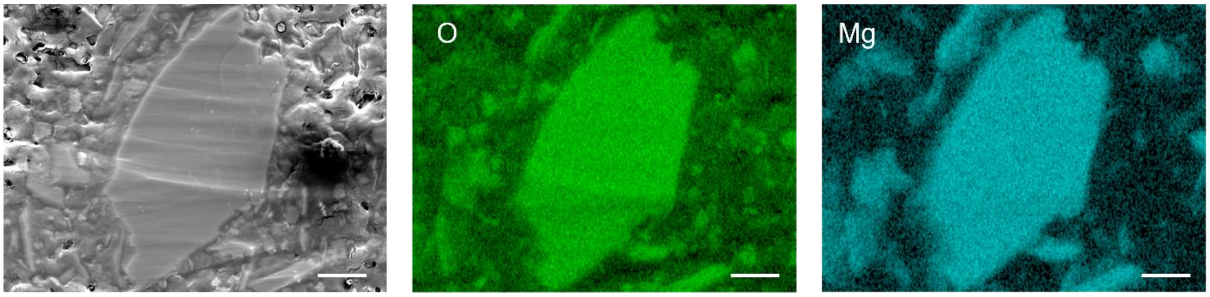

Supplementary Fig. S11. Cross-section SEM-EDS mapping of LCM-HC. Scale bars, 5 μm. Single crystal-like morphology was observed without pore and Mg^{2+} was evenly distributed within Li_2CO_3 particles, consistent with solid solution behavior.

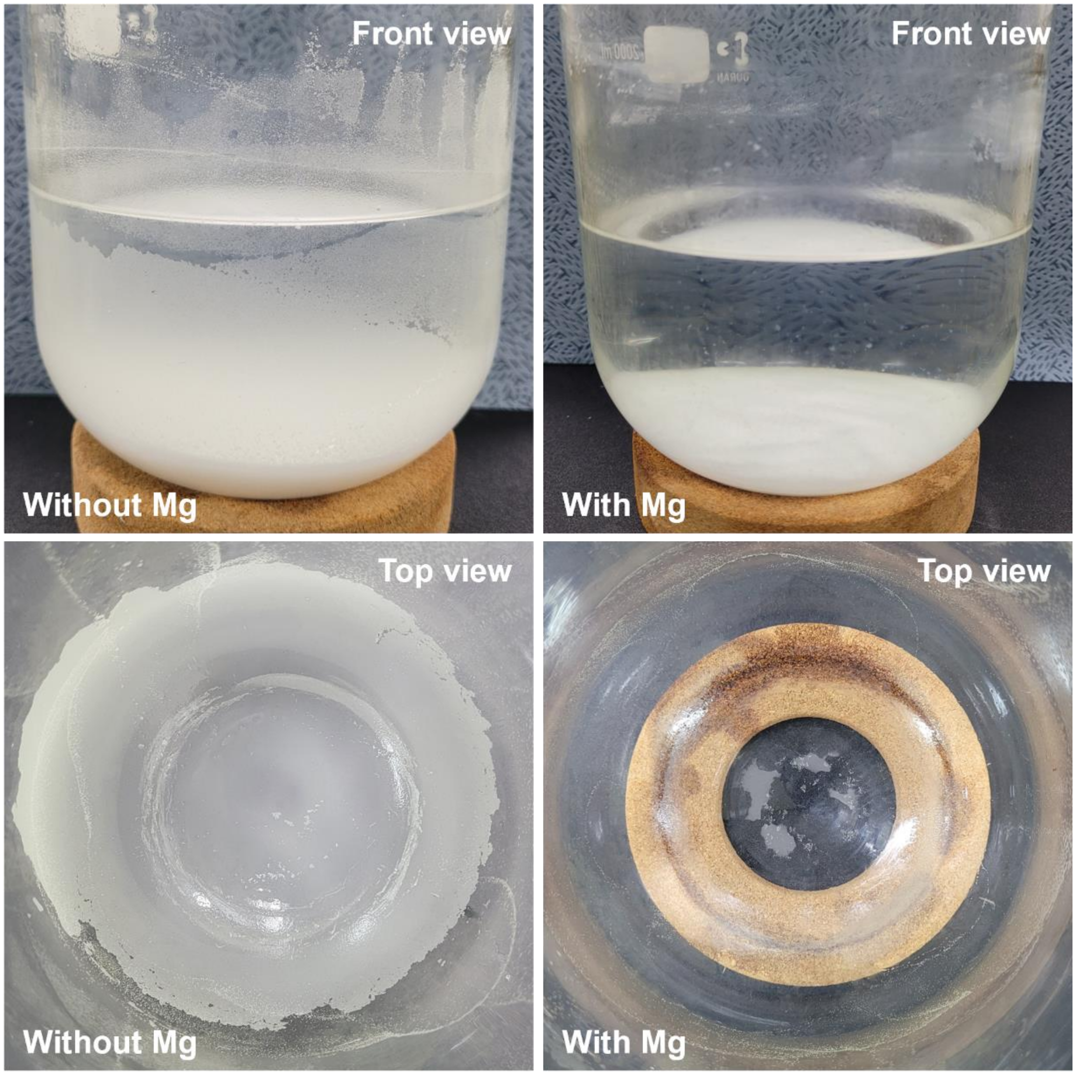

Supplementary Fig. S12. Lithium carbonate clogging test result according to residual Mg impurity. Mg impurity drastically alleviated Li_2CO_3 deposition by the seeding effect and morphology control. No deposition was observed with Mg while white Li_2CO_3 sediment on reactor wall was observed without Mg.

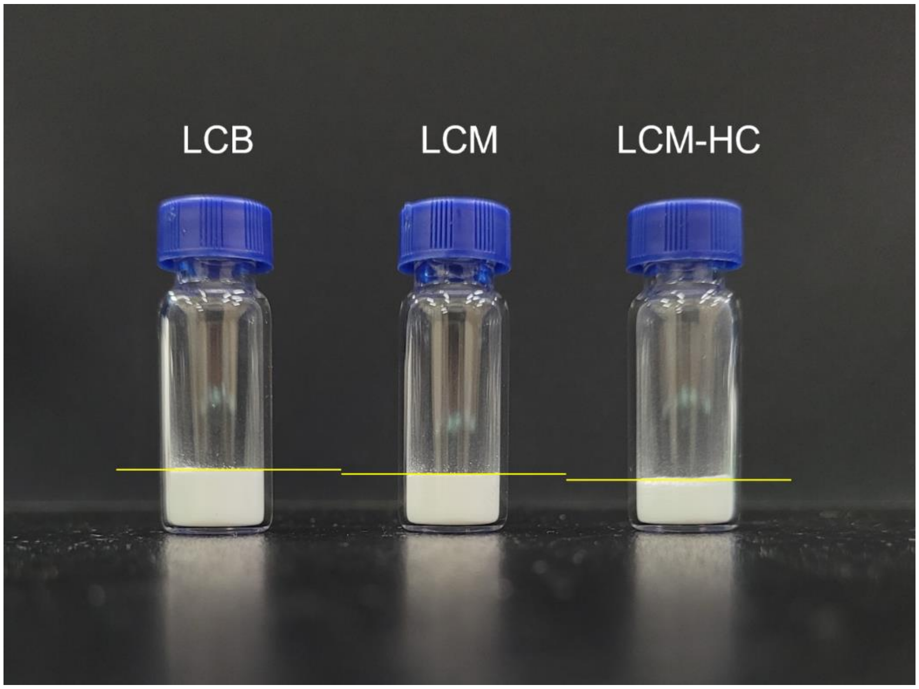

Supplementary Fig. S13. The filling ratio of cathode material synthesis furnace was tested by roughly comparing the tap density of lithium salts. The 0.3 g of lithium salts were placed into identical 2 ml vials. The tap density exhibited a slight increase with the higher Mg impurity content.

Supplementary Table S4. The inductive coupled plasma optical emission spectrometry results of cathode materials from various Li_2CO_3 with different Mg^{2+} concentrations. Mg^{2+} concentrations of 0.0, 0.3, and 0.9 g L^{-1} were denoted as Bare, LCD, and LCD-HC from LCB, LCM, and LCM-HC. All element contents are in mol% and normalized by the sum of Ni, Co, and Mn.

Mg concentration (g L^{-1})	Li	Ni	Co	Mn	Mg
0.0	1.052	0.600	0.200	0.200	N/D
0.1	1.009	0.600	0.200	0.201	0.005
0.3	1.021	0.600	0.200	0.200	0.016
0.5	1.005	0.599	0.201	0.200	0.032
0.7	1.010	0.600	0.199	0.200	0.035
0.9	0.993	0.599	0.201	0.201	0.036

N/D: not detected

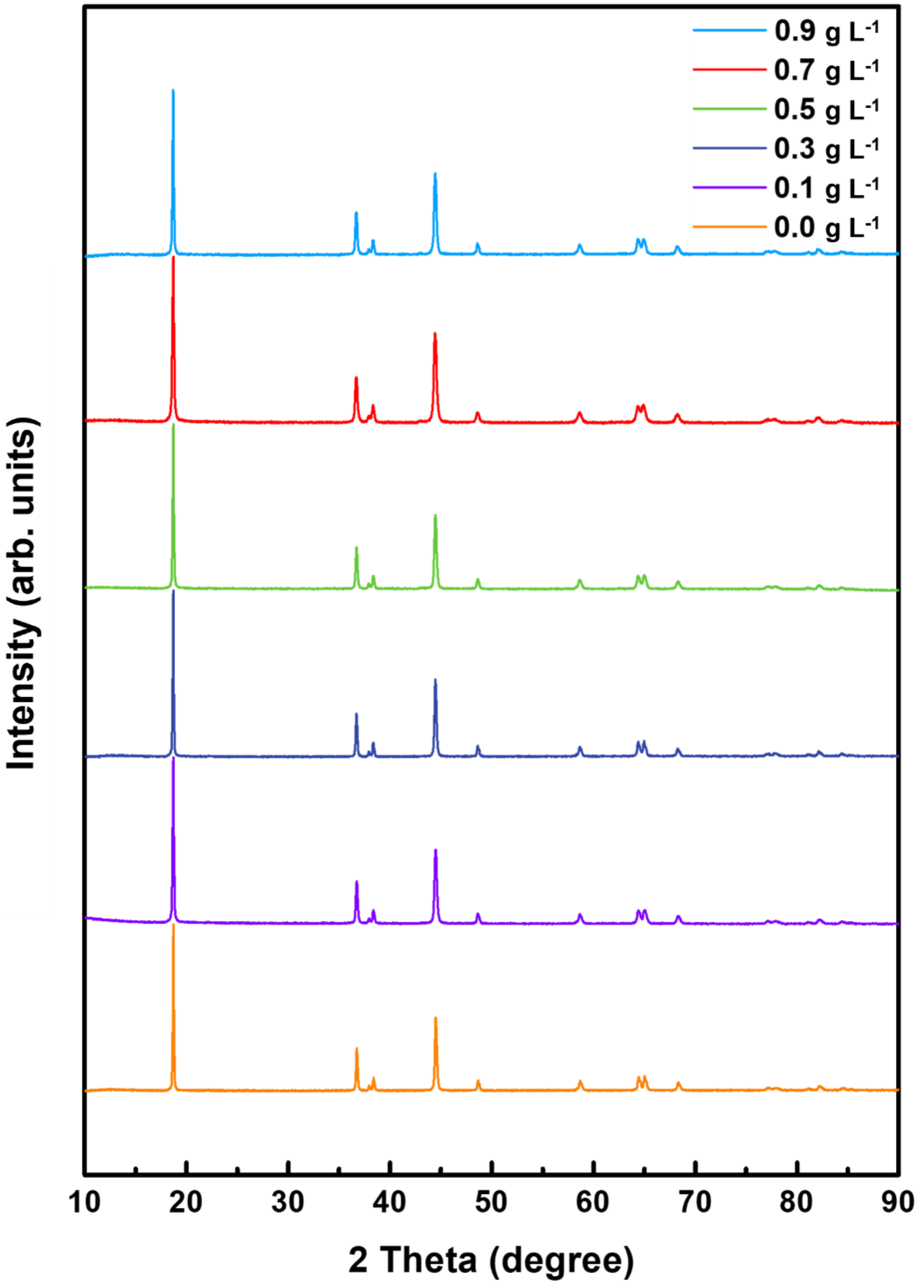

Supplementary Fig. S14. Comparison of XRD patterns of cathode materials from various Li_2CO_3 with different Mg^{2+} concentrations. Mg^{2+} concentrations of 0.0, 0.3, and 0.9 g L^{-1} were denoted as Bare, LCD, and LCD-HC from LCB, LCM, and LCM-HC.

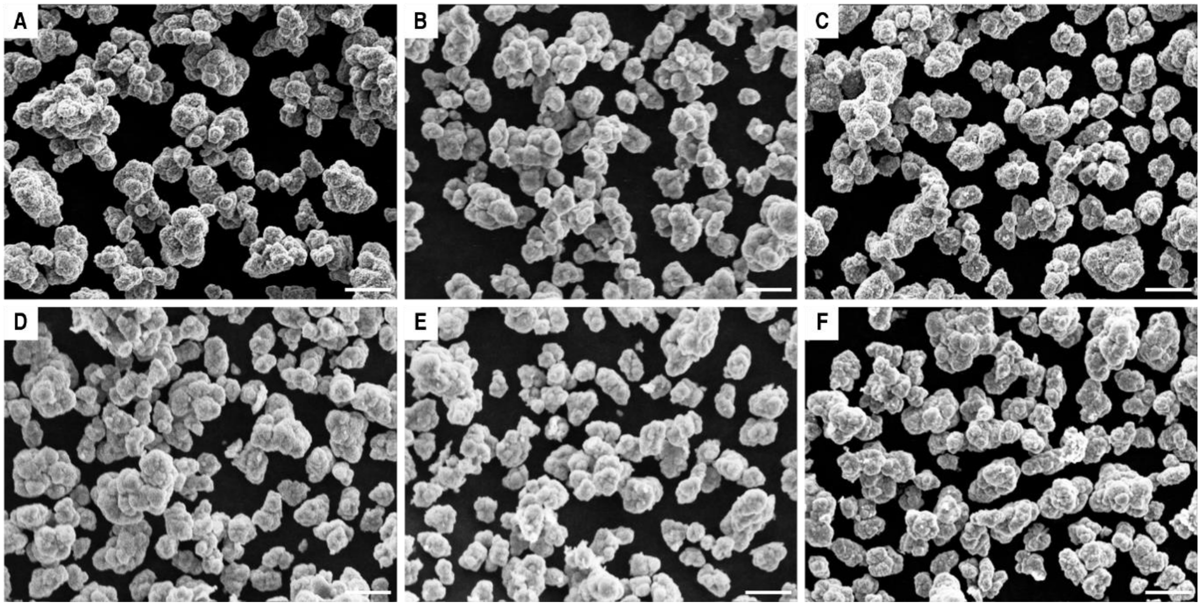

Supplementary Fig. S15. Comparison of SEM images of cathode materials from various Li_2CO_3 with different Mg^{2+} concentrations of 0.0 g L^{-1} (**A**), 0.1 g L^{-1} (**B**), 0.3 g L^{-1} (**C**), 0.5 g L^{-1} (**D**), 0.7 g L^{-1} (**E**), and 0.9 g L^{-1} (**F**). Mg^{2+} concentrations of 0.0, 0.3, and 0.9 g L^{-1} were denoted as Bare, LCD, and LCD-HC from LCB, LCM, and LCM-HC. Scale bars, 10 μm.

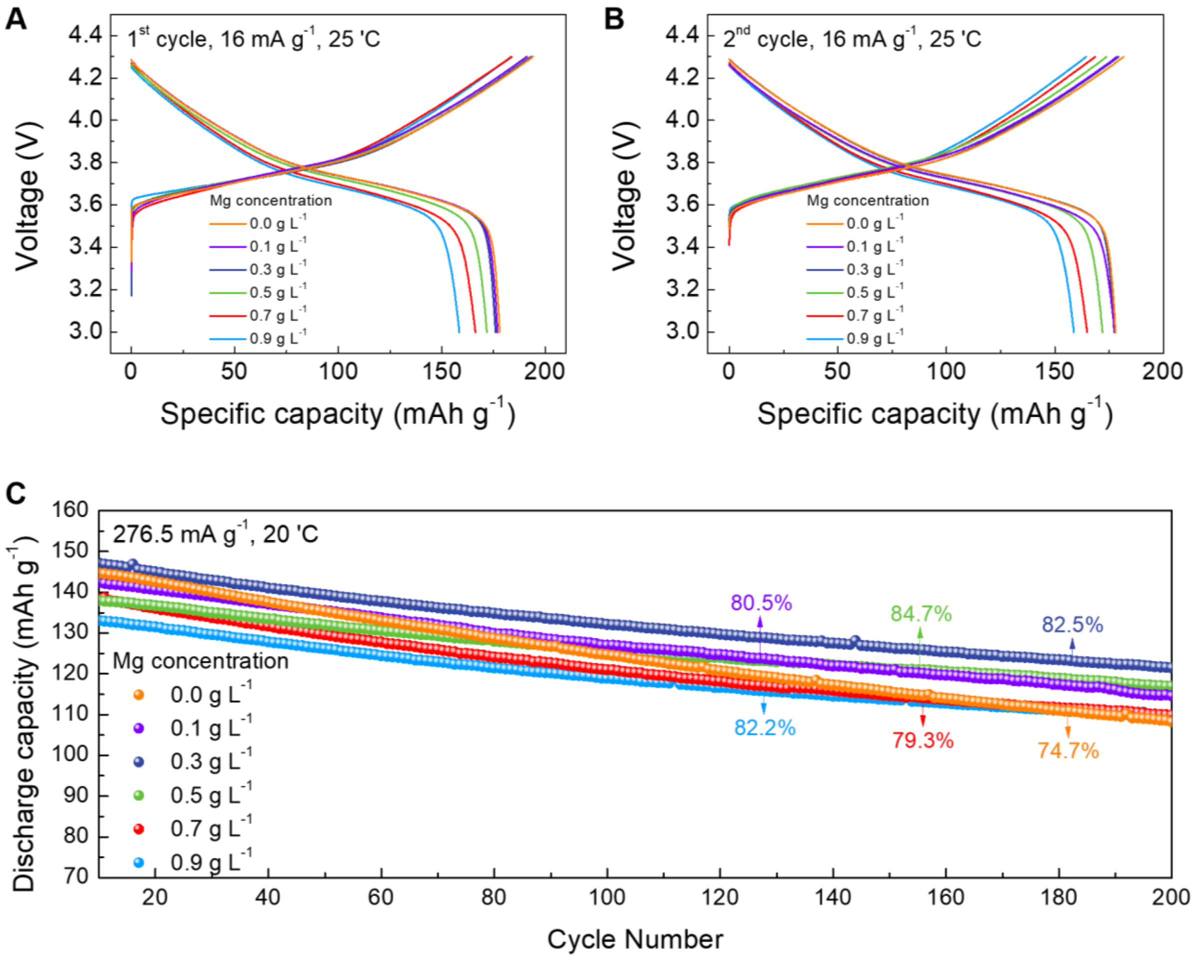

Supplementary Fig. S16. Electrochemical measurements of cathodes from various Li_2CO_3 with different Mg^{2+} concentrations in the reaction batch. Charge/discharge curves of various cathode materials with voltage rages of 3.0–4.3 V at a current density of 16 mA g^{-1}; first cycles (**A**), second cycles (**B**). (**C**) Capacity fading of these cathodes at a current density of 276.5 mA g^{-1} with a cutoff voltage of 4.3 V. The capacity retentions of these cathodes (Mg^{2+} concentrations of 0.0, 0.1, 0.3, 0.5, 0.7, and 0.9 g L^{-1}) were 74.7, 80.5, 82.5, 84.7, 79.3 and 82.2%, respectively. Mg^{2+} concentrations of 0.0, 0.3, and 0.9 g L^{-1} were denoted as Bare, LCD, and LCD-HC from LCB, LCM, and LCM-HC, respectively.

Supplementary Text for Supplementary Fig. S16.

The electrochemical measurements were implemented to compare performance of these cathodes. (**A**) and (**B**) show charge/discharge curves of first and second cycles at 16 mA g^{-1} in the voltage range of 3.0−4.3 V. The initial discharge capacities of cathodes (0.0−0.9 g L^{-1}) were 178.0, 176.9, 175.9, 171.8, 166.2, and 158.4 mAh g^{-1}, respectively. The initial capacities gradually decreased as the concentration of Mg doping increased. (**C**) shows that all of cathodes calcined with Mg^{2+} containing Li_2CO_3 demonstrated improved cyclability up to 200 cycles than the undoped cathode (denoted as “Bare”, 0.0 g L^{-1}), yet the loss of initial capacity turned out severely over 0.5 g L^{-1}.

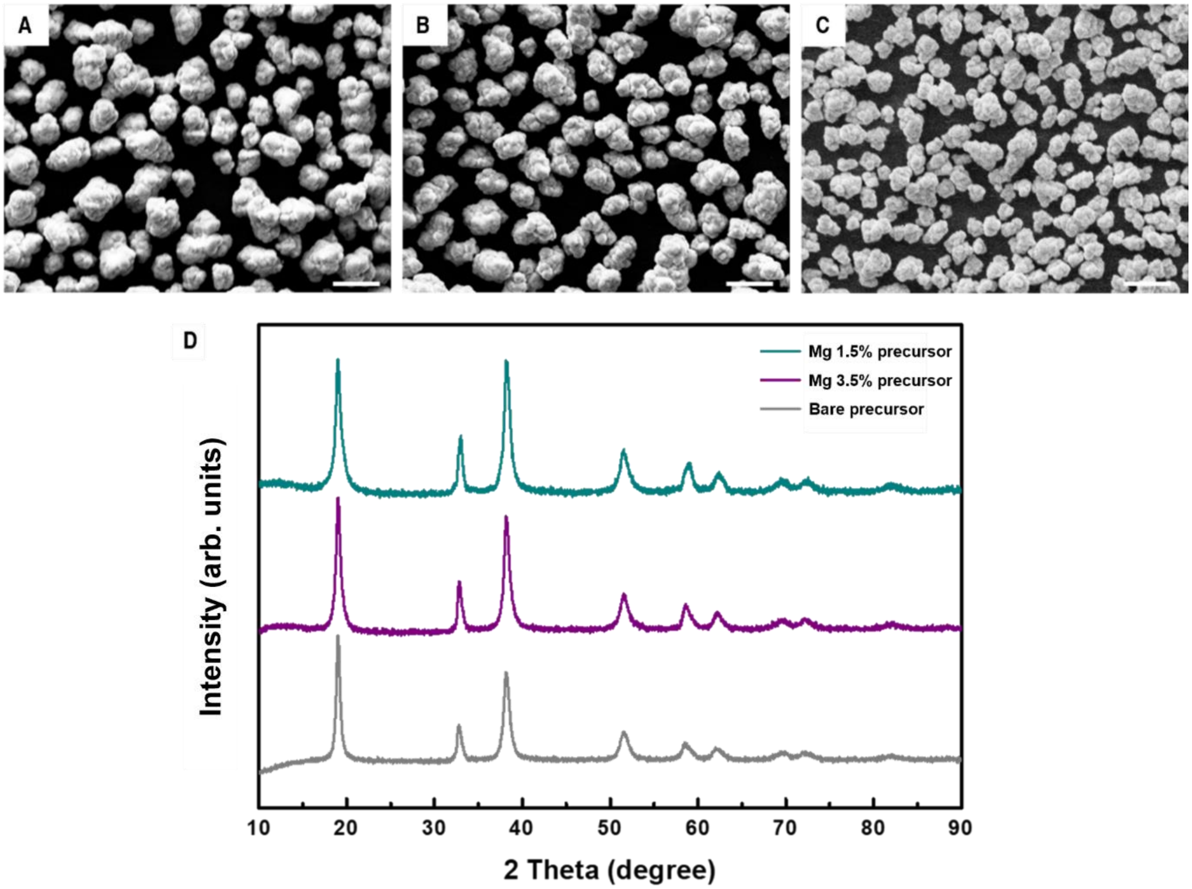

Supplementary Fig. S17. Transition metal hydroxide precursors synthesis and characterization. Comparison of SEM images of transition metal hydroxide precursors; Bare precursor (**A**), Mg 1.5% precursor (**B**), and Mg 3.5% precursor (**C**). (**D**) XRD patterns of transition metal hydroxide precursors. Scale bars, 10 μm.

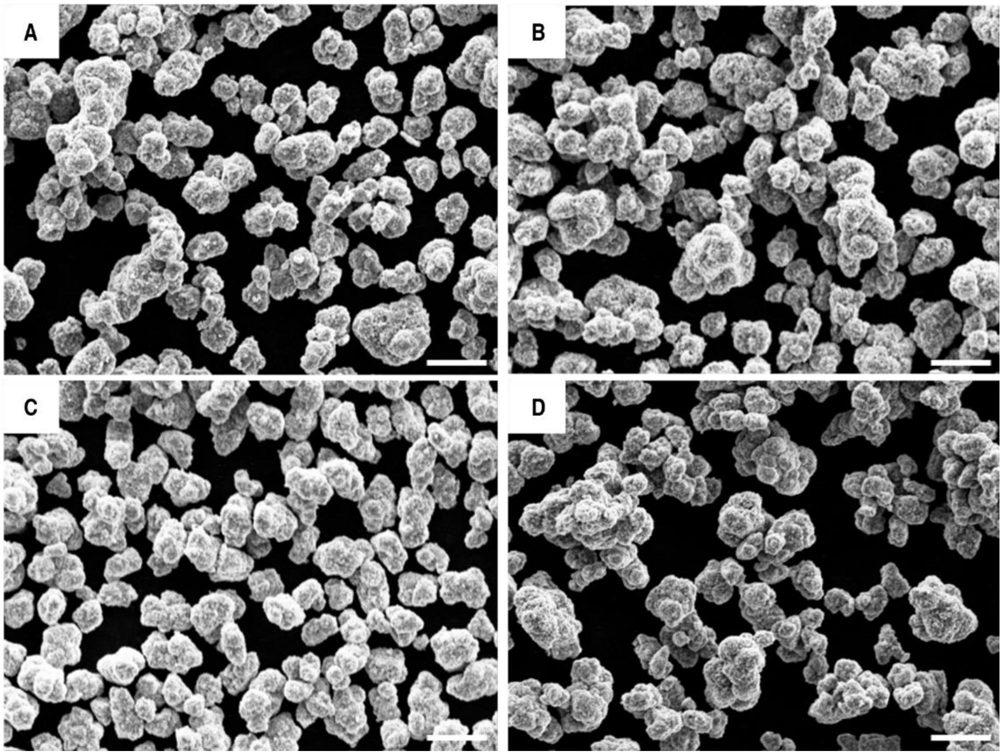

Supplementary Fig. S18. Comparison of SEM images of $LiNi_{0.6}Co_{0.2}Mn_{0.2}O_2$ cathode materials containing 1.5 mol% of Mg^{2+}; LCD (**A**), SSD (**B**), CPD (**C**), and Bare (**D**). Scale bars, 10 μm.

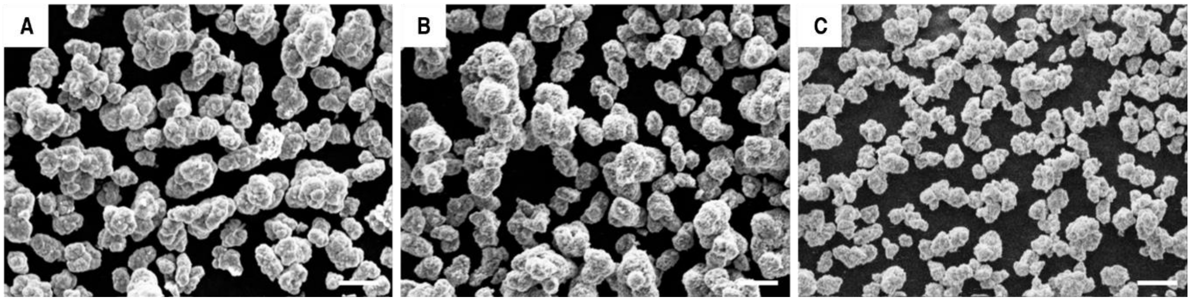

Supplementary Fig. S19. Comparison of SEM images of $LiNi_{0.6}Co_{0.2}Mn_{0.2}O_2$ cathode materials containing 3.5 mol% of Mg^{2+}; LCD-HC (**A**), SSD-HC (**B**), and CPD-HC (**C**). Scale bars, 10 μm.

Supplementary Table S5. The inductive coupled plasma optical emission spectrometry results of Bare precursor, Mg 1.5% precursor, Mg 3.5% precursor, Bare, LCD, SSD, CPD, LCD-HC, SSD-HC, and CPD-HC. All element contents are normalized by the sum of Ni, Co, and Mn.

**Atomic Ratio**	**Li**	**Ni**	**Co**	**Mn**	**Mg**
Bare precursor	N/D	0.600	0.201	0.199	N/D
Mg 1.5% precursor	N/D	0.601	0.202	0.197	0.016
Mg 3.5% precursor	N/D	0.600	0.200	0.200	0.034
Bare	1.052	0.600	0.200	0.200	N/D
LCD	1.021	0.600	0.200	0.200	0.016
SSD	1.035	0.599	0.200	0.201	0.014
CPD	1.043	0.600	0.202	0.198	0.015
LCD-HC	0.993	0.599	0.201	0.201	0.036
SSD-HC	1.003	0.599	0.200	0.201	0.034
CPD-HC	1.043	0.601	0.199	0.200	0.036

N/D: not detected

Supplementary Note S3:

The Mg^{2+} doped cathodes by the solid-state-doping method were produced by mixing separated $MgCO_3$ before calcination (**Supplementary Note S1**; solid-state-doping), denoted as SSD. The Mg^{2+} containing transition metal hydroxide precursors were prepared by coprecipitation method adding extra Mg^{2+} with 1.5 mol% (**Supplementary Note S1**). The transition metal hydroxide precursor had identical morphology and crystal structure with bare $Ni_{0.6}Co_{0.2}Mn_{0.2}(OH)_2$ hydroxide (**Supplementary Fig. S17**), and we synthesize another 1.5 mol% Mg doped NCM622 by coprecipitation method, named as CPD (**Supplementary Note S1**; coprecipitation-doping). All cathode materials were synthesized having similar particle morphology (**Supplementary Figs. S18 and S19**), and ICP-OES analysis confirmed that powders were within the target composition range as shown in **Supplementary Table S5**.

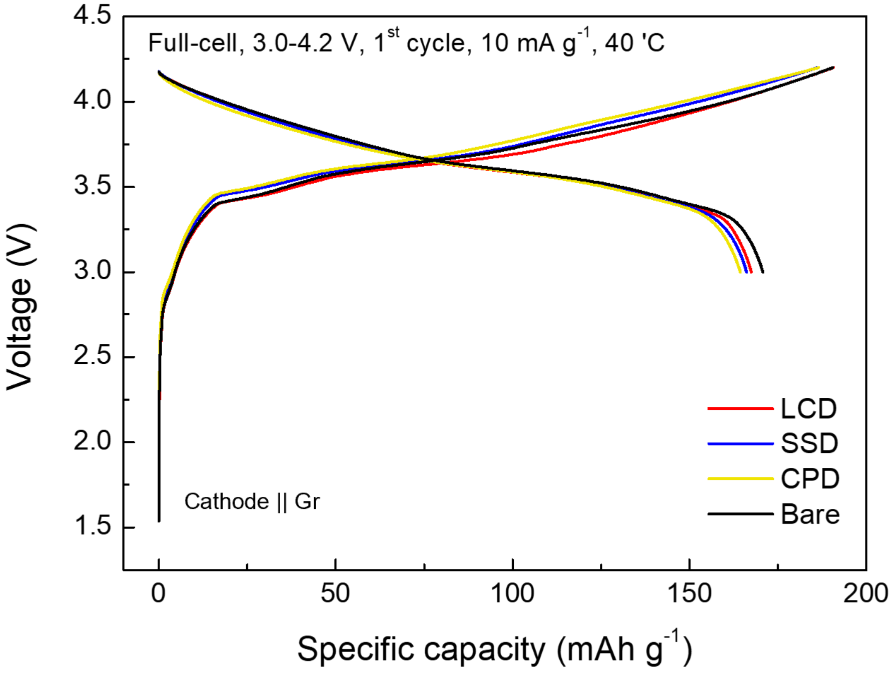

Supplementary Fig. S20. The full cell (graphite anode) charge/discharge curves of LCD, SSD, CPD, and Bare in the voltage range of 3.0–4.2 V (vs. graphite) at a current density of 10 mA g^{-1} at the formation cycle. The formation cycle was conducted after wetting at 1.5 V for 12 h.

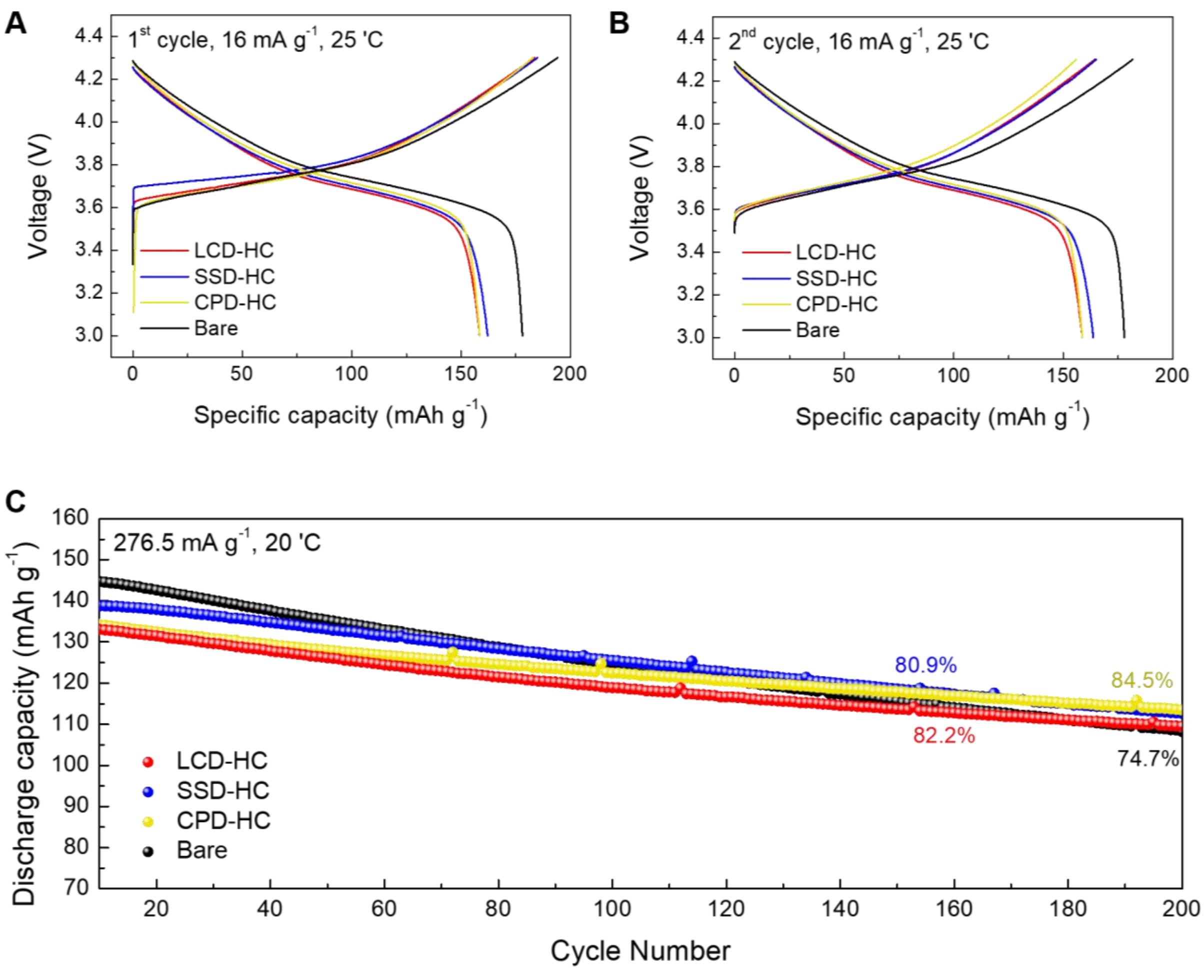

Supplementary Fig. S21. Electrochemical measurements of LCD-HC, SSD-HC, and CPD-HC comparing with Bare. Charge/discharge curves of LCD-HC, SSD-HC, CPD-HC, and Bare in the voltage range of 3.0−4.3 V at a current density of 16 mA g^{-1}; first cycles (**A**), second cycles (**B**). (**C**) Capacity fading of these cathodes at a current density of 276.5 mA g^{-1} with a cutoff voltage of 4.3 V. The capacity retentions of LCD-HC, SSD-HC, and CPD-HC were 82.2, 80.9, and 84.5%, respectively.

Supplementary Text for Supplementary Fig. S21.

With increasing Mg^{2+} concentration up to 3.5 %, the significant losses of initial capacities were observed for all samples (**A**) and (**B**). The initial discharge capacities of LCD-HC, SSD-HC, and CPD-HC were obtained as 158.5, 162.1, and 158.5 mAh g^{-1}, respectively, at a current density of 16 mA g^{-1}. (**C**) shows that LCD-HC, SSD-HC, and CPD-HC retained 82.2 %, 80.9%, and 84.5%, respectively, capacity retentions after 200 cycles at 276.5 mA g^{-1}, which were still greater than Bare (74.7%). Overall, Mg^{2+} doping itself enhanced cycling performance of the cathodes. In contrast, high concentration of Mg^{2+} doping (~3.5 mol%) led cathodes to loss initial capacity.

Supplementary Note S4:

The sources of lithium extraction are diverse, e.g., spodumene, brine, clay, sea water, and oilfield wastewater. The different sources contain varying level of impurities, in fact, the content of Li and impurities exhibit quite large variations, even in spodumene mined at the same mine. To address this issue, many lithium extraction companies already have implemented a two-step purification process denoted as “Mg elimination step” (the first step) and “Ca elimination step” (the second step), as depicted in **Fig. 1**. In detail, the majority of Mg and the trace impurities of Fe, Mn, Al, etc. are eliminated at Mg elimination step. Precipitation of polyvalent ions (≥3+) occurs at lower pH levels, allowing for the complete elimination of trace impurity metals before the removal of Mg^{2+}. After the Mg elimination step, both the original Ca^{2+} ions and the additional Ca^{2+} ions from $Ca(OH)_2$ are eliminated along with any remaining Mg^{2+} during the Ca elimination step. Since Ca^{2+} precipitates at a lower pH compared to Mg^{2+}, the adjustment of the refining process load allows for the retention of Mg^{2+} if desired. Consequently, even with variations in impurity levels due to different source types and qualities, a consistent level of refined products can be achieved by modifying the refining process. This approach holds true for controlling the concentration of the Mg impurity as well.

For better understanding, the following examples of our pilot plant test results are given:

Supplementary Table S6. The composition of Li_2SO_4 solution at each purification steps.

Sample	Li (g L^{-1})	S (g L^{-1})	**Mg (g L^{-1})**	K (g L^{-1})
Leachate	11.00	31.98	**0.246**	0.202
Mg elimination step	11.14	26.12	**0.108**	0.201
Ca elimination step	11.46	25.45	**N/D**	0.200

N/D: not detected

This table presents the composition of the Li_2SO_4 solution at each purification step, highlighting the concentrations of Mg^{2+} at various stages. Following acid roasting, the leachate contains Mg^{2+} at a concentration of 0.246 g L^{-1}. During the Mg elimination step, the Mg^{2+} concentration decreases to 0.108 g L^{-1} as it reacts with $Ca(OH)_2$, resulting in the formation of $Mg(OH)_2$ precipitate. The remaining Mg^{2+} concentration after the Mg elimination step can be easily

controlled by adjusting the amount of $Ca(OH)_2$ used. In conventional processes used for producing *battery-grade* Li_2CO_3, Mg impurities are thoroughly eliminated through a subsequent Ca elimination step, as indicated in this table. However, in our work, there is no requirement to introduce additional Na_2CO_3 for eliminating any remaining Mg impurities. By simply adjusting the amount of Na_2CO_3 used to solely eliminate Ca^{2+}, the same amount of Mg impurities can still be retained after the Ca elimination step, owing to differences in precipitation pH. Therefore, regardless of the initial concentration of Mg^{2+} in the leachate, the impurity concentration of Mg^{2+} can be regulated through a straightforward adjustment of the refining process.

The following example demonstrates how the refining process can be adjusted to maintain a specific concentration of Mg^{2+}:

Supplementary Table S7. Acid roasting impurity test. The composition of Li_2SO_4 leachate and solution after Mg elimination step by H_2SO_4 input.

H_2SO_4 input (g)	Sample	Li (g L^{-1})	S (g L^{-1})	**Mg (g L^{-1})**	K (g L^{-1})
20	Leachate	11.01	31.14	**0.268**	0.026
	Mg elimination step	11.07	25.04	**0.102**	0.021
40	Leachate	11.77	28.53	**3.16**	0.033
	Mg elimination step	11.24	22.71	**0.127**	0.031
80	Leachate	11.72	25.45	**4.97**	0.058
	Mg elimination step	11.31	24.81	**0.108**	0.052

When the amount of sulfuric acid used to extract lithium hard rock is changed, it results in a corresponding alteration in the amount of impurities present during the acid roasting step. The adjustment of the sulfuric acid quantity directly affects the concentration of impurities in the acid roasting process. In this manner, leachates with varying levels of impurities were prepared, and the purification process was modified to maintain a constant concentration of Mg^{2+}. By adjusting the refining process accordingly, the amount of Mg^{2+} present after the Mg elimination step could be controlled and maintained at a consistent level, regardless of the initial impurity content.

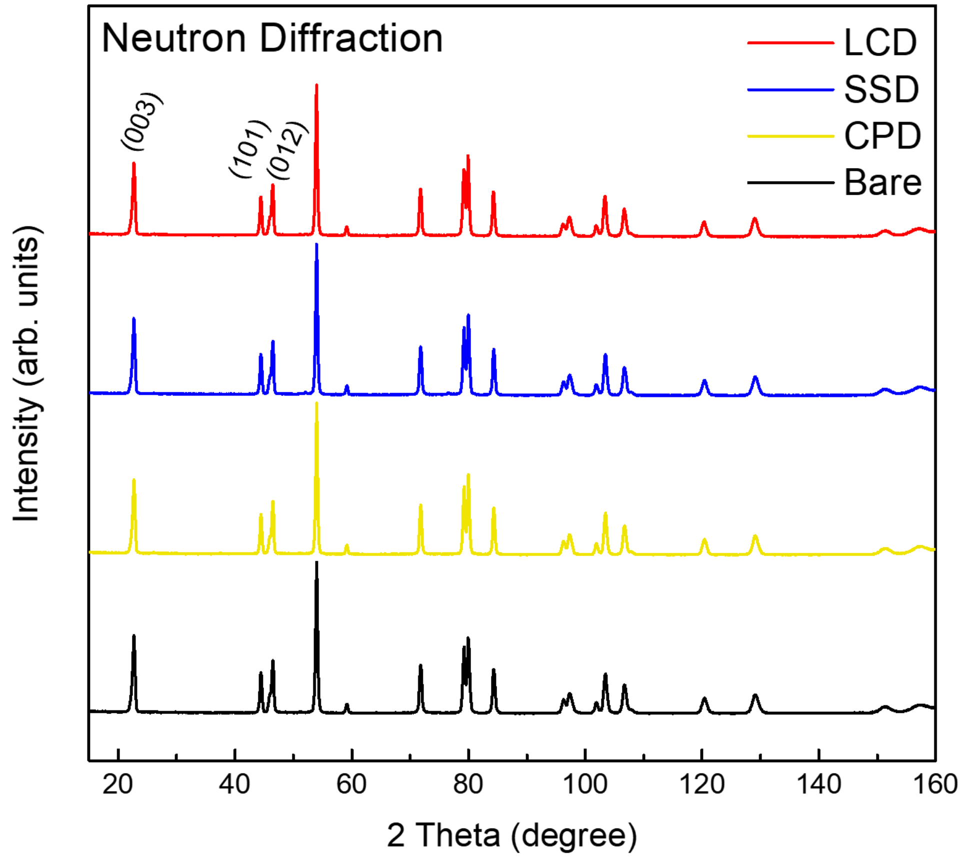

Supplementary Fig. S22. ND patterns of LCD, SSD, CPD, and Bare.

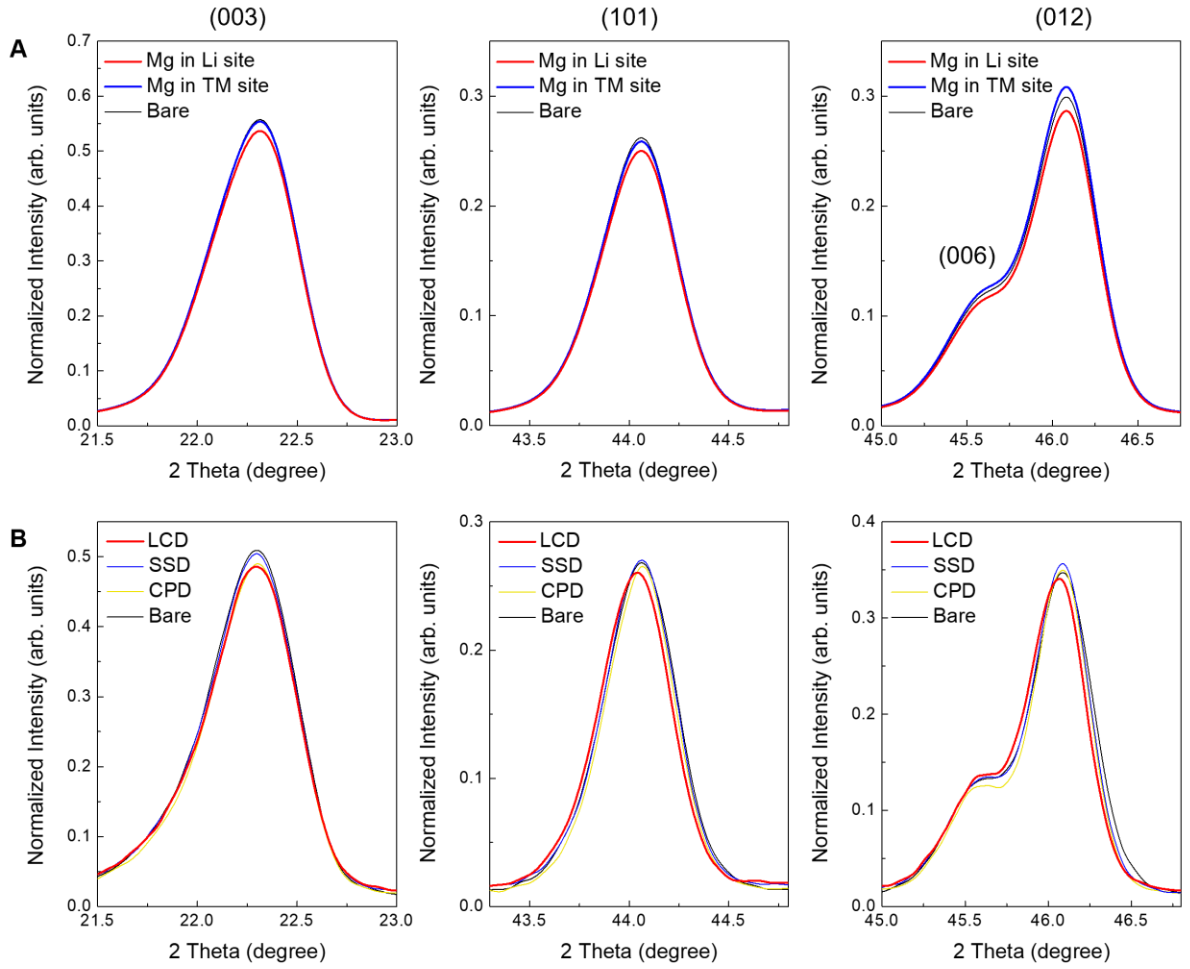

Supplementary Fig. S23. Whole pattern shapes of simulated (**A**) and observed (**B**) ND patterns comparison. ND simulation was carried out using thermal and position factors from Bare's refinement result (pristine NCM622), and the 1.5 mol% Mg^{2+} doping into Li site or TM site (exchanged with Ni ions) was systematically simulated without changing the lattice or positions of atoms.

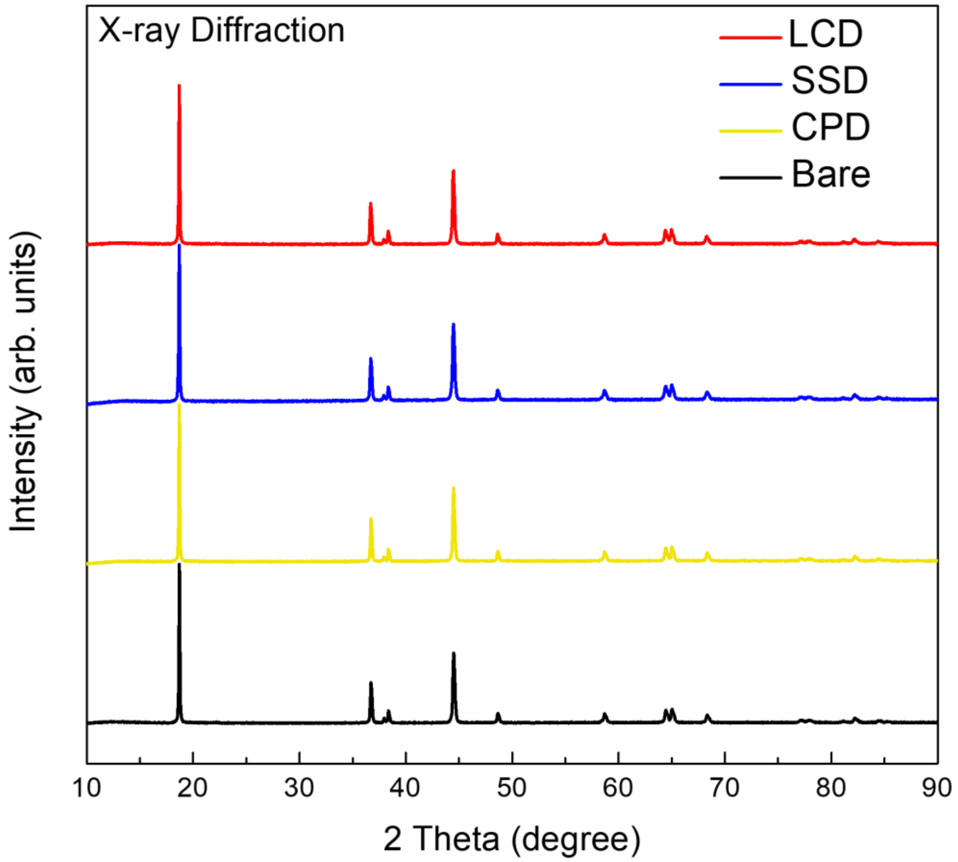

Supplementary Fig. S24. XRD patterns of LCD, SSD, CPD, and Bare.

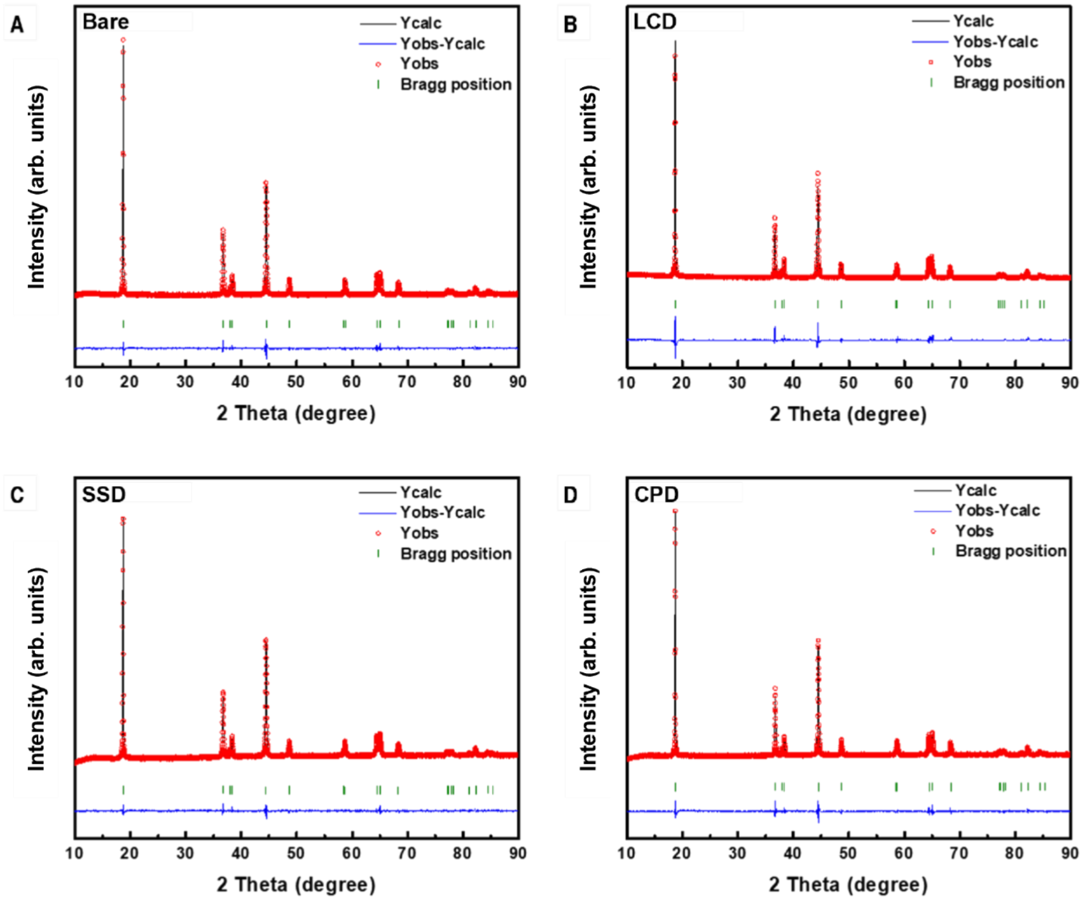

Supplementary Fig. S25. XRD Rietveld refinement fitting data of Bare (**A**), LCD (**B**), SSD (**C**), and CPD (**D**).

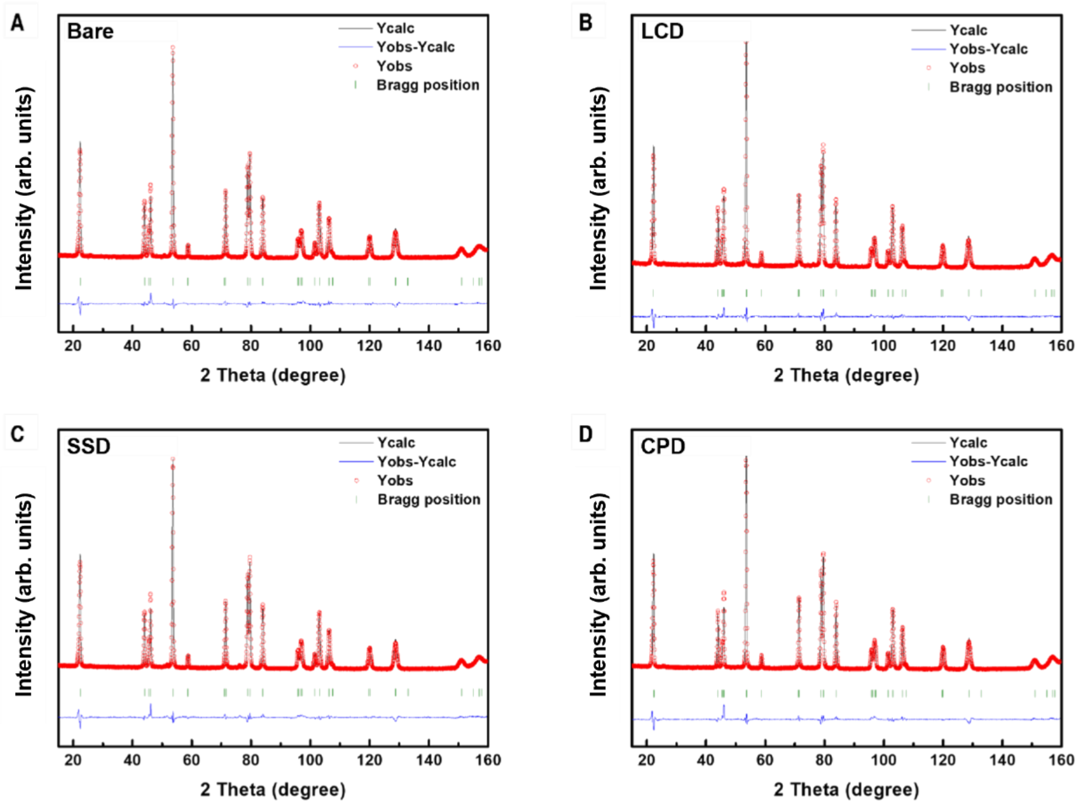

Supplementary Fig. S26. ND Rietveld refinement fitting data of Bare (**A**), LCD (**B**), SSD (**C**), and CPD (**D**).

Supplementary Table S8. XRD Refinement parameters of Bare, LCD, SSD, and CPD.

Parameter	Bare	LCD	SSD	CPD
c (Å)	14.2157(38)	14.2307(85)	14.2236(35)	14.2222(62)
a (Å)	2.86600(26)	2.87160(96)	2.86722(24)	2.86596(42)
Volume ($Å^3$)	101.123(2)	101.626(3)	101.266(2)	101.167(2)
O z position	0.2408(2)	0.2416(6)	0.2418(2)	0.2409(4)
R_{wp} (%)	1.41	5.07	1.26	1.72

Supplementary Table S9. ND Refinement parameters of Bare.

Bare	x	y	z	B	Occ
Li (3a)	0	0	0	1.25(52)	0.972(25)
Ni (3a)	0	0	0	1.25(52)	0.028(25)
Ni (3b)	0	0	0.5	0.45(52)	0.572(25)
Co (3b)	0	0	0.5	0.45(52)	0.2
Mn (3b)	0	0	0.5	0.45(52)	0.2
Li (3b)	0	0	0.5	0.45(52)	0.028(25)
O (6c)	0	0	0.2415(19)	0.85(52)	1

Ni_{Li} (%) = 2.82, R_p (%) = 2.66, R_{wp} (%) = 3.86

Supplementary Table S10. ND Refinement parameters of LCD.

**LCD**	x	y	z	B	Occ
Li (3a)	0	0	0	1.37(41)	0.963(23)
Ni (3a)	0	0	0	1.37(41)	0.023(23)
Mg (3a)	0	0	0	1.37(41)	0.013(23)
Ni (3b)	0	0	0.5	0.57(41)	0.561(23)
Co (3b)	0	0	0.5	0.57(41)	0.2
Mn (3b)	0	0	0.5	0.57(41)	0.2
Li (3b)	0	0	0.5	0.57(41)	0.036(23)
Mg (3b)	0	0	0.5	0.57(41)	0.003(23)
O (6c)	0	0	0.2416(12)	0.97(41)	1

occ (Mg_{3a}) / occ (Mg_{tot}) (%) = 84.2, Ni_{Li} (%) = 2.30, R_p (%) = 2.49, R_{wp} (%) = 3.50

Supplementary Table S11. ND Refinement parameters of SSD.

**SSD**	x	y	z	B	Occ
Li (3a)	0	0	0	1.30(57)	0.965(31)
Ni (3a)	0	0	0	1.30(57)	0.026(31)
Mg (3a)	0	0	0	1.30(57)	0.009(31)
Ni (3b)	0	0	0.5	0.50(57)	0.575(31)
Co (3b)	0	0	0.5	0.50(57)	0.2
Mn (3b)	0	0	0.5	0.50(57)	0.2
Li (3b)	0	0	0.5	0.50(57)	0.035(31)
Mg (3b)	0	0	0.5	0.50(57)	0.005(31)
O (6c)	0	0	0.2416(18)	0.90(57)	1

occ (Mg_{3a}) / occ (Mg_{tot}) (%) = 65.0, Ni_{Li} (%) = 2.63, R_p (%) = 2.87, R_{wp} (%) = 4.08

Supplementary Table S12. ND Refinement parameters of CPD.

**CPD**	x	y	z	B	Occ
Li (3a)	0	0	0	1.27(53)	0.964(30)
Ni (3a)	0	0	0	1.27(53)	0.026(30)
Mg (3a)	0	0	0	1.27(53)	0.010(30)
Ni (3b)	0	0	0.5	0.47(53)	0.575(30)
Co (3b)	0	0	0.5	0.47(53)	0.2
Mn (3b)	0	0	0.5	0.47(53)	0.2
Li (3b)	0	0	0.5	0.47(53)	0.036(30)
Mg (3b)	0	0	0.5	0.47(53)	0.005(30)
O (6c)	0	0	0.2415(18)	0.87(53)	1

occ (Mg_{3a}) / occ (Mg_{tot}) (%) = 65.6, Ni_{Li} (%) = 2.57, R_p (%) = 2.79, R_{wp} (%) = 4.04

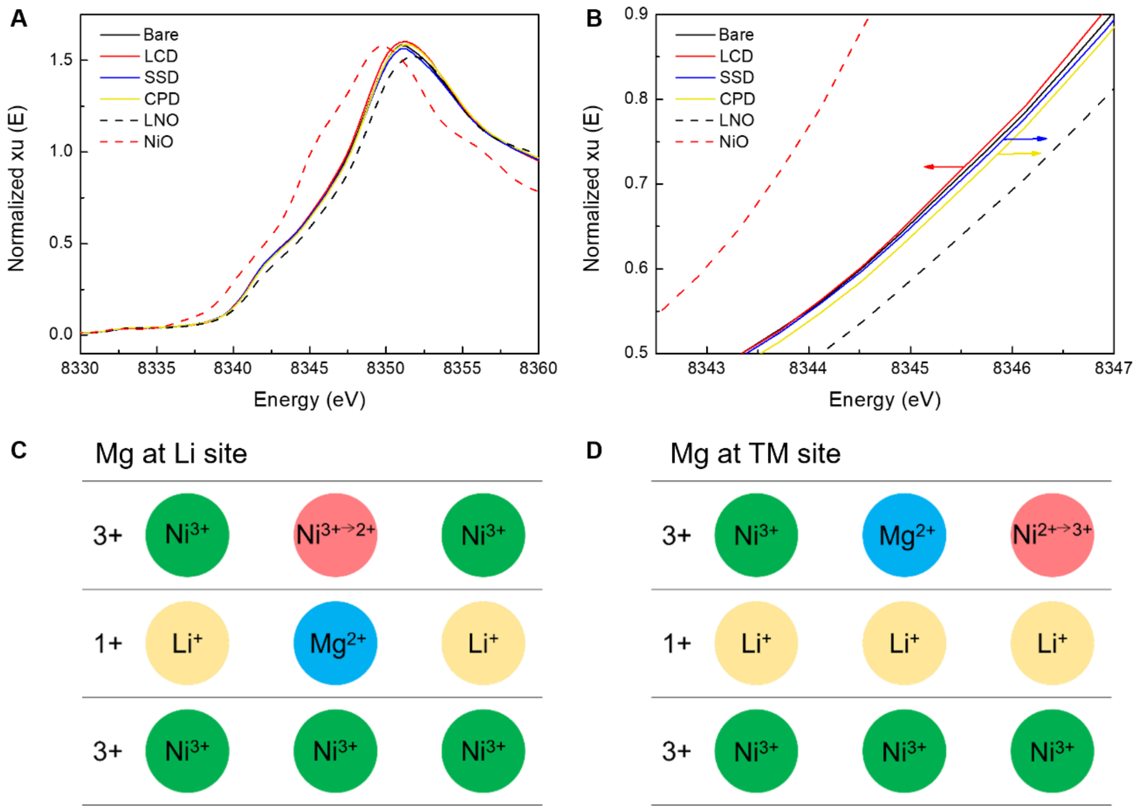

Supplementary Fig. S27. XAS analysis of cathode materials. (**A**) Ni K-edge X-ray absorption (XAS) analysis were conducted. (**B**) Enlarged edge region shows that only Ni in LCD is more reduced than Bare while SSD and CPD are more oxidized. The Ni shows different redox behavior by the position of Mg^{2+}. When Mg^{2+} locates at Li site (Mg_{Li}^{+}), (**C**) Ni reduces by charge compensation whereas (**D**) Ni oxidizes when Mg^{2+} locates at TM site (Mg_{TM}^{-}). This results strongly support doping site-selectivity of Mg depending on the synthetic route.

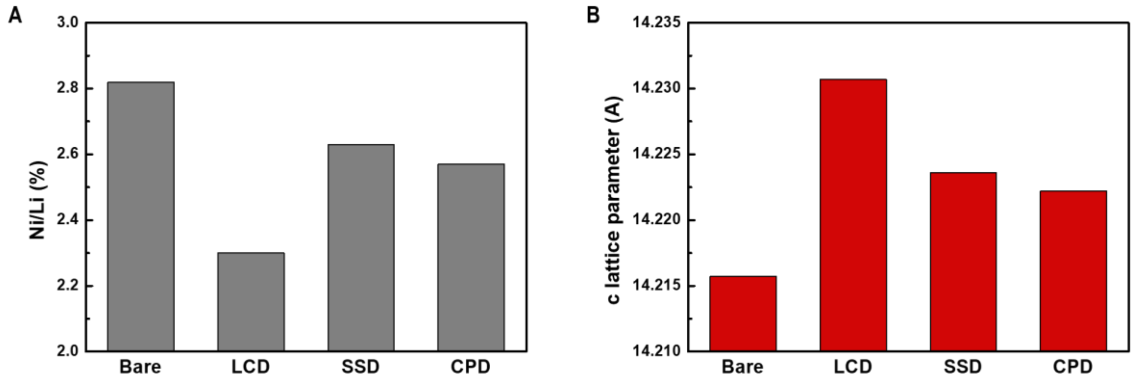

Supplementary Fig. S28. Quantitative atomic occupancy and structural analysis via ND and XRD Rietveld refinement. ND Rietveld refinement result of cation mixing (**A**) and XRD Rietveld refinement result of *c* lattice parameters (**B**).

Supplementary Text for Supplementary Fig. S28.

(**A**) The combined refinement results proves that the cation mixing ratio (Ni/Li%) of doped samples were decreased, indicating that the structural integrity of the crystal structure was improved. (**B**) However, all Mg^{2+} doped cathode materials showed increased *c* lattice parameters compared to that of Bare (14.2157(38) Å). Especially, LCD showed the largest *c* lattice parameter increasement to 14.2307(85) Å, and SSD and CPD increased *c* lattice parameters to 14.2236(35) and 14.2222(62) Å, respectively. This lattice change trend is also in good agreement with the preference for Mg-doped sites; The more divalent Mg ions exist in the Li slab, the greater the *c*-axis increase due to interlayer repulsion[3,4]. The improvement in rate capability and the slight increase in initial coulombic efficiency (ICE) in LCD (**Fig. 3**) are suggested to be a result of the increased *c* lattice parameter, which enhances Li^+ diffusion kinetics[5].

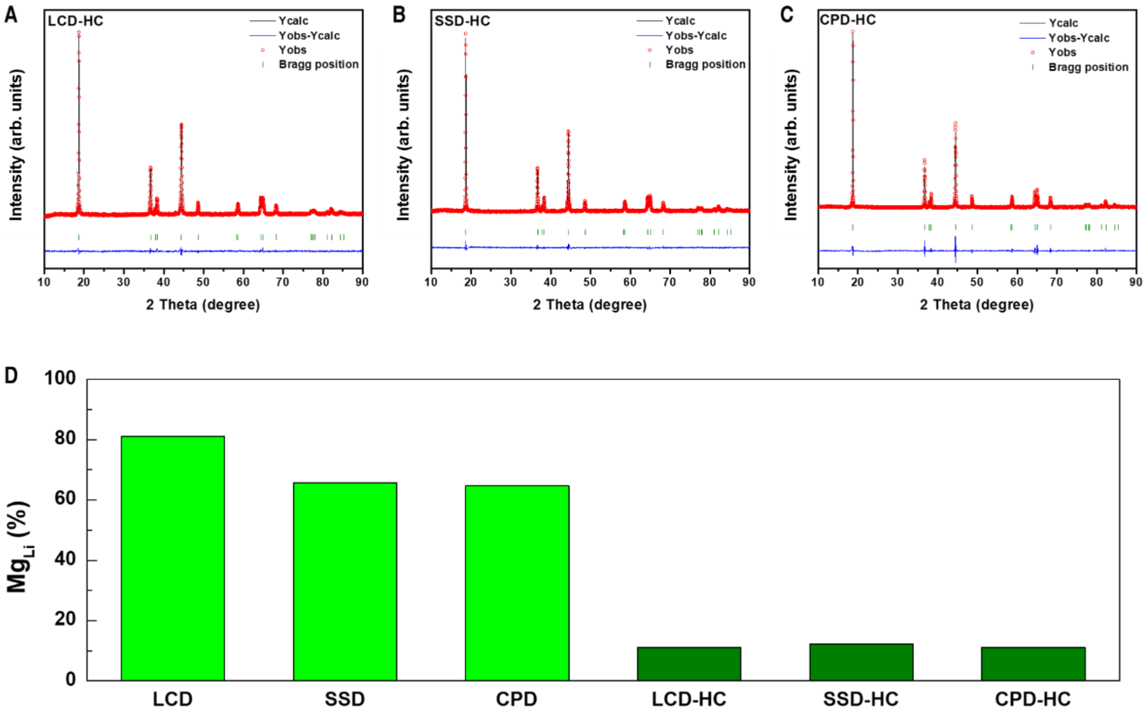

Supplementary Fig. S29. XRD Rietveld refinement fitting data of LCD-HC (**A**), SSD-HC (**B**), and CPD-HC (**C**). (**D**) Comparison of occupancy ratios of Mg in Li site of these comparable samples with the samples containing ~1.5 mol% of Mg^{2+} (LCD, SSD, and CPD from ND Rietveld refinement).

Supplementary Table S13. XRD Refinement parameters of LCD-HC, SSD-HC, and CPD-HC.

Atom (occ%)	Position	LCD-HC	SSD-HC	CPD-HC
Li1	3a	0.965(2)	0.971(2)	0.981(4)
Ni1	3a	0.030(2)	0.025(2)	0.015(4)
Mg1	3a	0.004(2)	0.004(2)	0.004(4)
Ni2	3b	0.534(2)	0.540(2)	0.550(4)
Co2	3b	0.2	0.2	0.2
Mn2	3b	0.2	0.2	0.2
Li2	3b	0.035(2)	0.029(2)	0.019(4)
Mg2	3b	0.032(2)	0.029(2)	0.032(4)
O	6c	1	1	1
occ (Mg_{3a}) / occ (Mg_{tot}) (%)		11.11	12.12	11.11
R_{wp} (%)		1.21	1.25	2.12

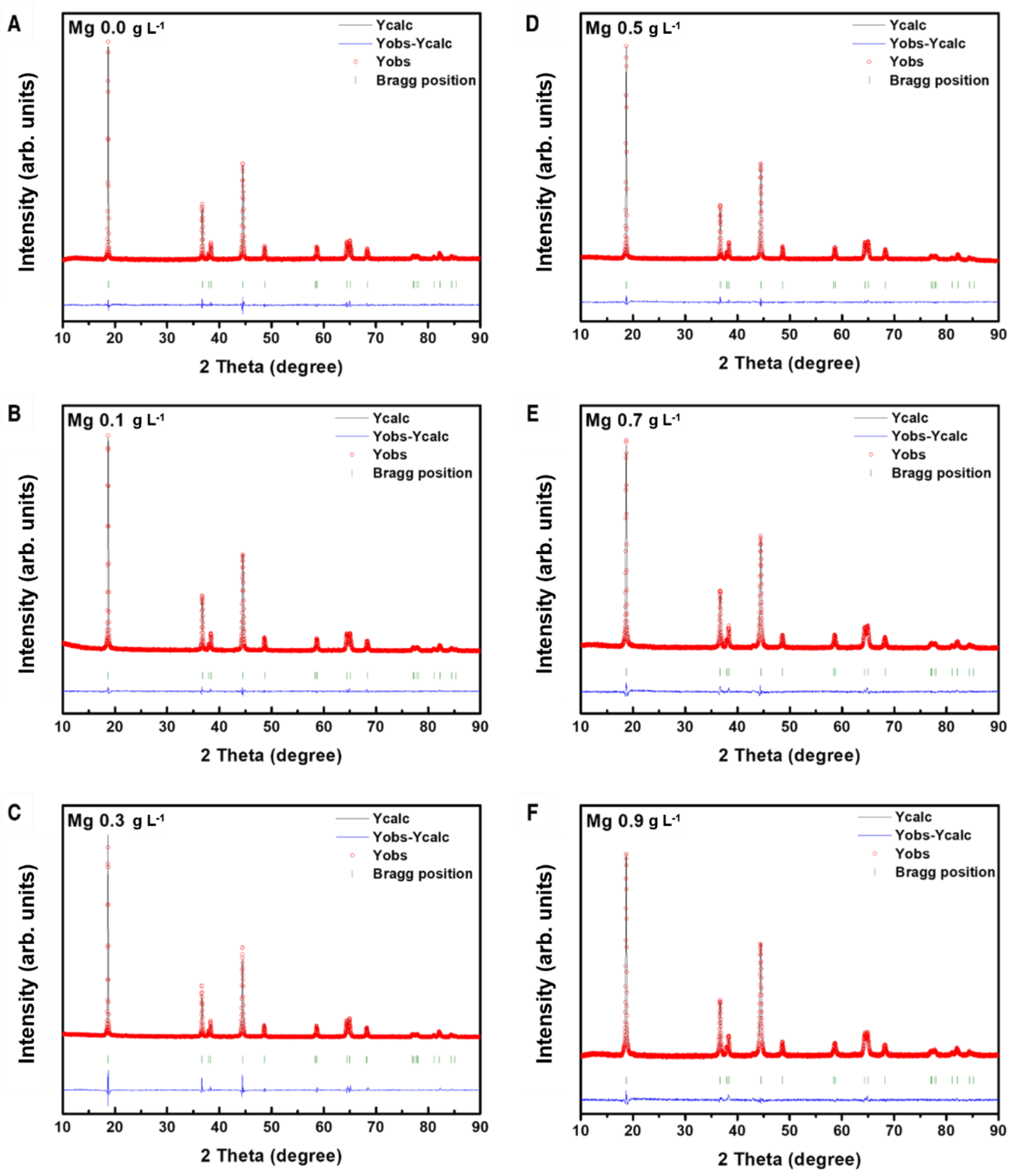

Supplementary Fig. S30. XRD Rietveld refinement fitting data of cathode materials from various Li_2CO_3 with different Mg^{2+} concentrations. XRD Rietveld refinement fitting data of cathode materials with Mg2+ concentration of 0.0 (**A**), 0.1 (**B**), 0.3 (**C**), 0.5 (**D**), 0.7 (**E**), and 0.9 g L^{-1} (**F**). Mg^{2+} concentrations of 0.0, 0.3, and 0.9 g L^{-1} were denoted as Bare, LCD, and LCD-HC from LCB, LCM, and LCM-HC.

Supplementary Table S14. XRD Refinement parameters of cathode materials from various Li_2CO_3 with different Mg^{2+} concentrations (Mg-0, Mg-1, Mg-3, Mg-5, Mg-7, and Mg-9 indicate cathode materials with Mg^{2+} concentrations of 0.0, 0.1, 0.3, 0.5, 0.7, and 0.9 g L^{-1}, respectively). Mg^{2+} concentrations of 0.0, 0.3, and 0.9 g L^{-1} were denoted as Bare, LCD, and LCD-HC from LCB, LCM, and LCM-HC. For better comparison, XRD occupancy analysis was conducted for Bare and LCD as well.

**Atom (occ%)**	**Position**	**Mg-0**	**Mg-1**	**Mg-3**	**Mg-5**	**Mg-7**	**Mg-9**
Li1	3a	0.984(2)	0.979(2)	0.968(6)	0.976(4)	0.961(4)	0.965(2)
Ni1	3a	0.016(2)	0.016(2)	0.018(6)	0.020(4)	0.034(4)	0.030(2)
Mg1	3a	0	0.005	0.013(6)	0.003(4)	0.004(4)	0.004(2)
Ni2	3b	0.584(2)	0.584(2)	0.579(6)	0.548(4)	0.531(4)	0.534(2)
Co2	3b	0.2	0.2	0.2	0.2	0.2	0.2
Mn2	3b	0.2	0.2	0.2	0.2	0.2	0.2
Li2	3b	0.016(2)	0.016(2)	0.018(6)	0.024(4)	0.039(4)	0.035(2)
Mg2	3b	0	0	0.003(6)	0.0287(4)	0.031(4)	0.032(2)
O	6c	1	1	1	1	1	1
occ (Mg_{3a}) / occ (Mg_{tot}) (%)		-	100.00	81.25	12.35	9.57	11.11
R_{wp} (%)		1.41	2.85	5.07	3.20	3.34	1.21

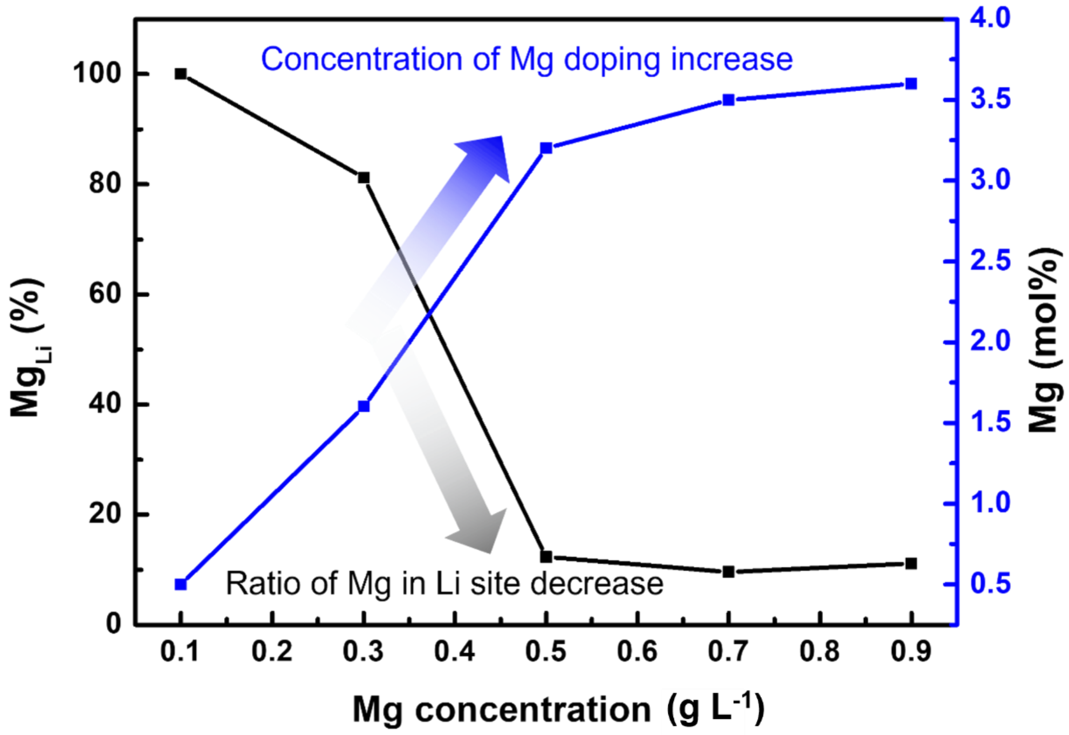

Supplementary Fig. S31. Comparison of Rietveld refinement results (occupancy ratios of Mg^{2+} in Li^{+} site) and ICP-OES results (concentrations of Mg^{2+} in cathode materials) of cathode materials from various Li_2CO_3 with different Mg^{2+} concentrations. The ratio of Mg^{2+} in Li^{+} site sharply decreased over 0.3 g L^{-1} of Mg^{2+} impurity concentration in a Li_2CO_3-synthesis batch as concentration of Mg^{2+} in cathode materials increases over 3 mol%. Mg^{2+} concentrations of 0.0, 0.3, and 0.9 g L^{-1} were denoted as Bare, LCD, and LCD-HC from LCB, LCM, and LCM-HC.

Supplementary Note S5:

In addition to electrochemical study (**Supplementary Fig. S21**), further investigation of high concentration (3.5 mol%) Mg^{2+} doped cathode materials were conducted (**Supplementary Fig. S29A−C and Table S13**). The Mg^{2+} occupancy ratio to Li site greatly reduced to ~10% at high concentration Mg^{2+} doped cathode materials (**Supplementary Fig. S29D**). It is known that Mg^{2+} prefer to enter Li slab when the amount of Mg^{2+} below 2 mol%[6]. The tendency between doping amount and the Li site occupancy is well described by the series of structural analysis (**Supplementary Fig. S14**). The Rietveld fitting data and parameters of cathode materials made of various Mg^{2+} containing Li_2CO_3 (0.0 to 0.9 g L^{-1} of Mg^{2+} impurity) are shown in **Supplementary Fig. S30 and Table S14**. It was found that as concentration of Mg^{2+} doping increased, the occupancy ratio of Mg^{2+} in Li site decreased (**Supplementary Fig. S31**). It is noticeable that the Li site occupancy drops to ~10% above the doping concentration of ~3 mol%. This result highly support that Li site occupancy ratio lowered at 3.5 mol% Mg^{2+} doped cathode materials. Thus, high concentration Mg^{2+} doped cathode materials exhibited poor electrochemical performances (**Supplementary Fig. S21**) disrupting Li^+ diffusion and diminishing initial capacity by excess Mg^{2+}.

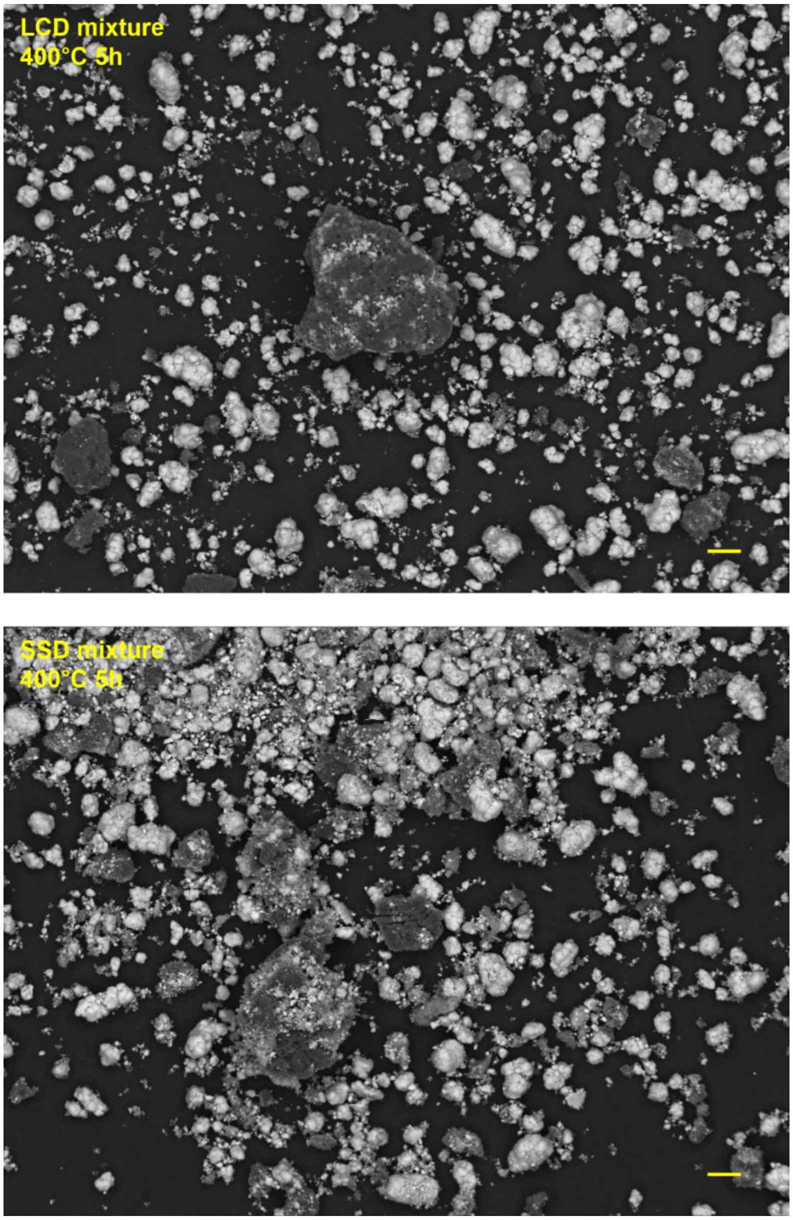

Supplementary Fig. S32. SEM backscattered electron (BSE) image of LCD and SSD mixtures. Particles with larger atomic number (Z) elements emit higher intensity of BSE; here, bright particles are (Ni, Co, Mn)-O, and dark particles are Li_2CO_3. The BSE images show that the lithium and transition metal precursors were homogeneously mixed. More transition metal precursor covers Li_2CO_3 particles at SSD mixture than LCD mixture, due to the secondary particle morphology of LCB in SSD mixture. Scale bars; 10 μm.

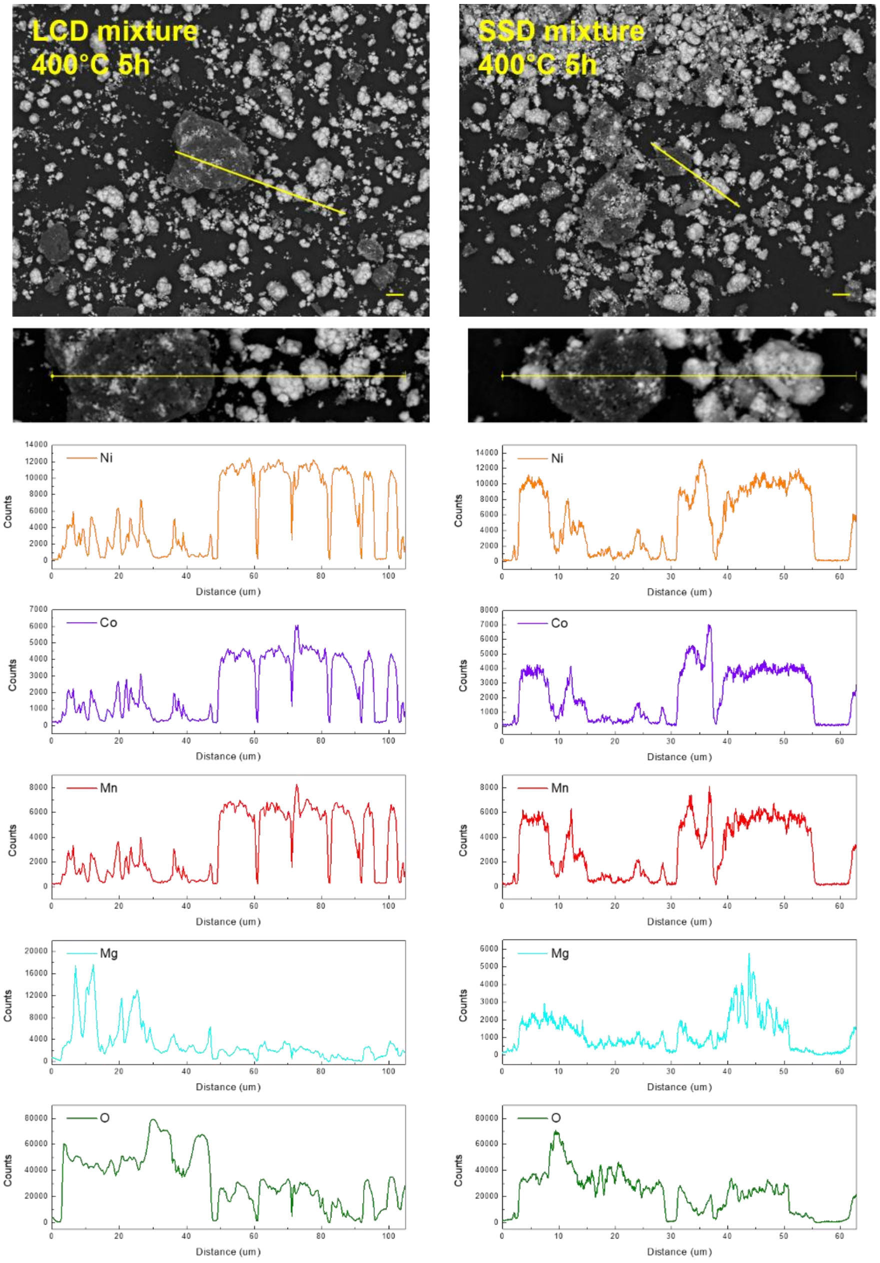

Supplementary Fig. S33. SEM-EDS line scan images of LCD and SSD mixtures. In LCD mixture, high Ni, Co, and Mn signal was detected at transition metal oxide particles, but negligible Mg signal. However, high Mg signal was detected at Li_2CO_3 particle that Mg impurity containing Li_2CO_3 (LCM) still hold Mg element inside. On the other hand, in SSD mixture, high Mg signal was detected at transition metal oxide particles with high Ni, Co, and Mn signal. The $MgCO_3$ in SSD mixture was reacted with transition metal oxide producing (Ni, Co, Mn, *Mg*)-O. Scale bars; 10 μm.

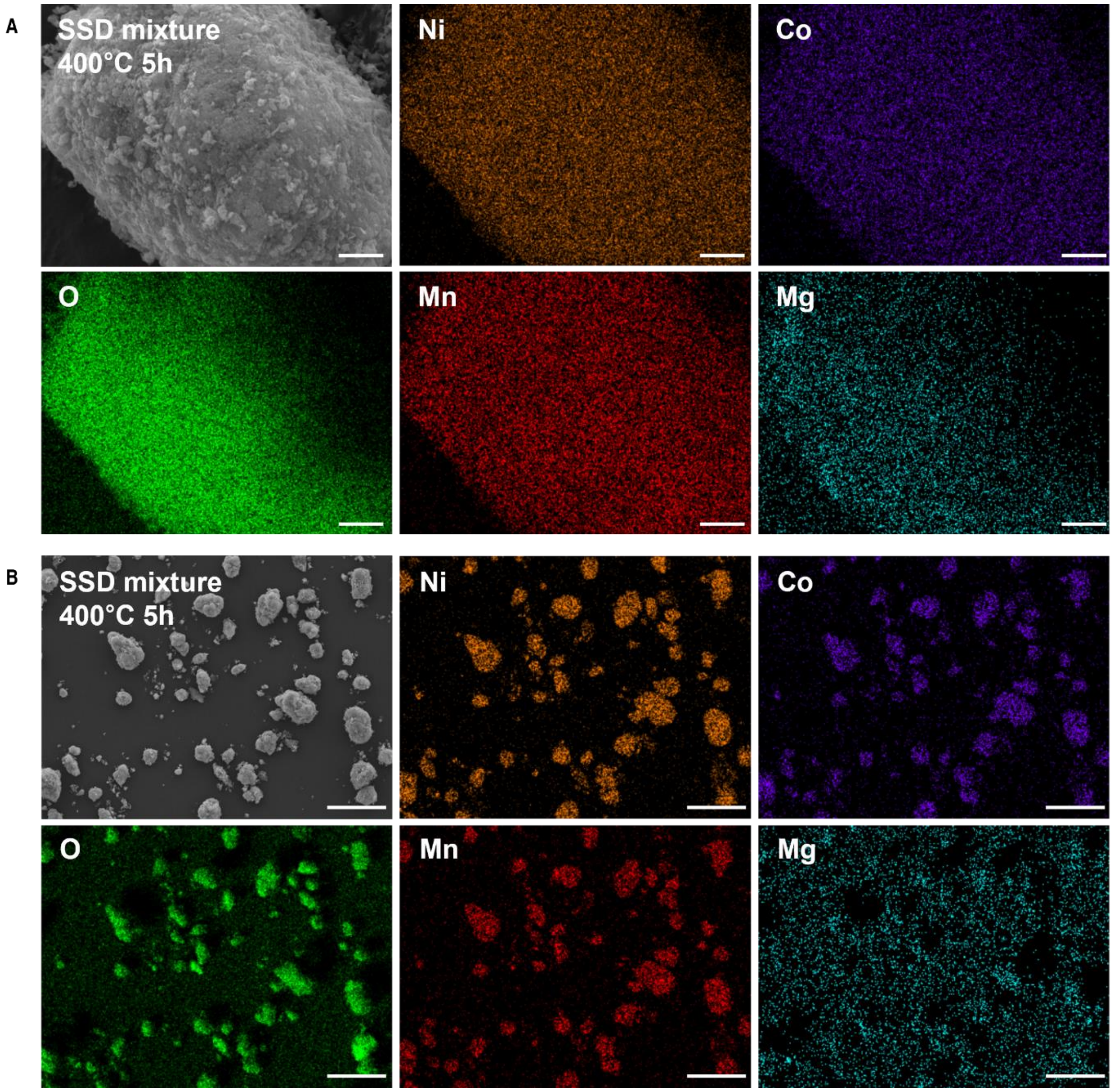

Supplementary Fig. S34. SEM-EDS images of SSD mixture (Bare precursor + LCB + $MgCO_3$) calcinated at 400 °C for 5 h. (**A**) Obvious Mg insertion into TM precursor particle was detected. Scale bars; 1 μm. (**B**) Low magnitude SEM-EDS images of SSD mixture. Mg dispersion throughout whole particle area was detected. Scale bars; 20 μm.

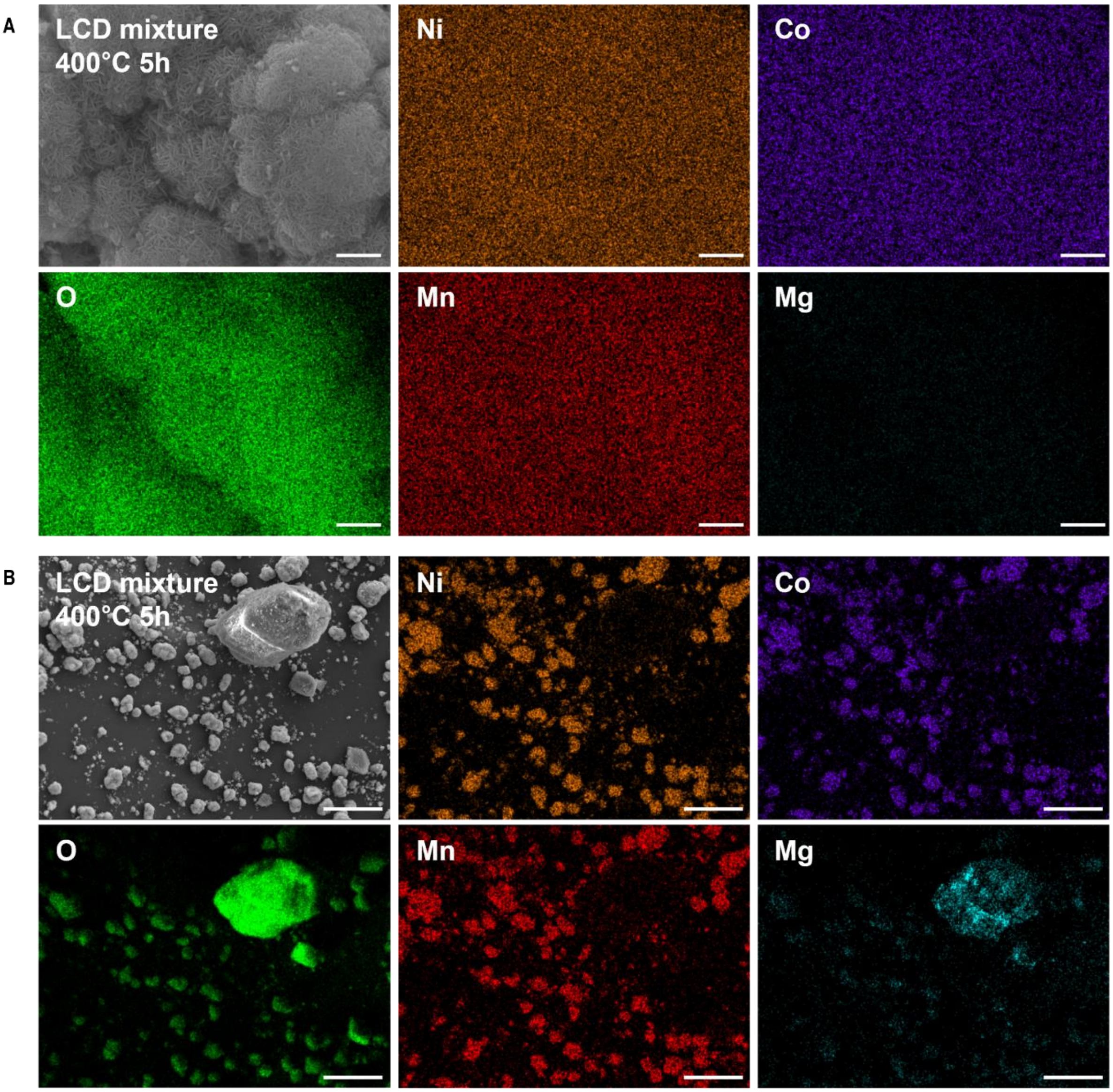

Supplementary Fig. S35. SEM-EDS images of LCD mixture (Bare precursor + LCM) calcinated at 400 °C for 5 h. (**A**) Negligible Mg insertion into TM precursor particle was detected. Scale bars; 1 µm. (**B**) Low magnitude SEM-EDS images of LCD mixture. Negligible Mg insertion into TM precursor particles while unmelted LCM holding Mg inside were detected. Scale bars; 20 µm.

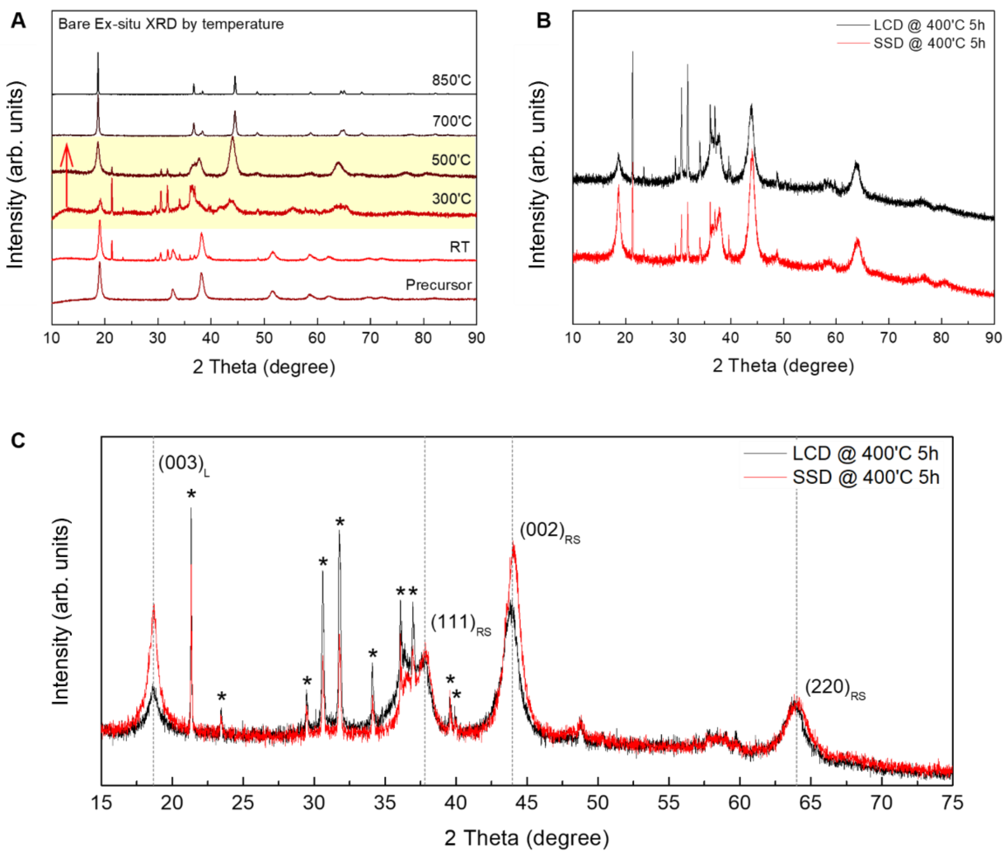

Supplementary Fig. S36. XRD analysis of intermediate phases. (**A**) Ex-situ XRD analysis of Bare by temperature. (**B**) XRD patterns of intermediate LCD and SSD mixtures calcinated at 400 °C for 5 h. (**C**) Overlayed XRD patterns with peak indices. The peak assignments of $()_L$, $()_{RS}$, and * refer to the layered, rocksalt, and lithium carbonate structures, respectively.

Supplementary Text for Supplementary Fig. S36.

The noticeable difference of peak intensity was observed at the peak indices of $(003)_L$ and $(002)_{RS}$. The ex-situ XRD analysis of Bare (**Supplementary Fig. S36a**, yellowish region between 300 and 500 °C) shows that the increase of peak intensity of $(003)_L$ and $(002)_{RS}$ indicates the improved crystallinity of intermediated phases. Increased peak intensity at SSD mixture compared to LCD mixture shows crystallinity improvement of Li_x(Ni, Co, Mn, *Mg*)O_2 (Li-poor rocksalt phase) and (Ni, Co, Mn, *Mg*)–O (rocksalt phase) which were assigned to $(003)_L$ and $(002)_{RS}$, respectively[7]. The presence of Mg in the intermediate phases of SSD would give an enhanced structural stability due to the bond dissociation energy difference. The bond dissociation energies of Mg–O and Ni–O are 394 kJ mol^{-1} and 391 kJ mol^{-1}, respectively[8], and the larger bond dissociation energy of Mg–O gave additional crystallinity improvement at Li_x(Ni, Co, Mn, *Mg*)O_2 and (Ni, Co, Mn, *Mg*)–O compared to Li_x(Ni, Co, Mn)O_2 and (Ni, Co, Mn)–O.

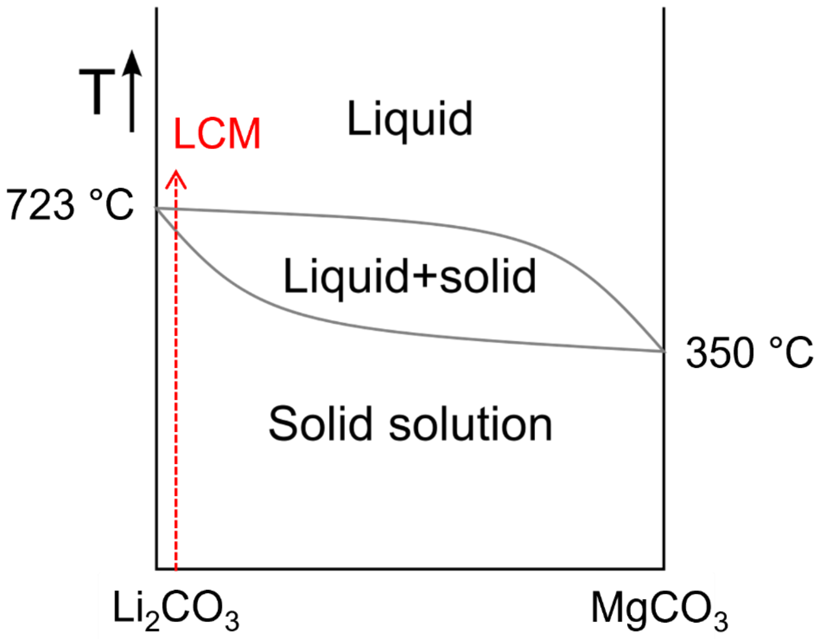

Supplementary Fig. 37. The conceptual phase diagram of solid solution LCM. The dilute $MgCO_3$ in Li_2CO_3 solid solution is expected to closely follow the melting behavior of the primary phase of Li_2CO_3.

Supplementary Table S15. CAPEX breakdown of commercial brine process and cost reduction less purifying Mg element.

Brine Process

CAPEX*		Cost (M$)	Cost reduction (M$)
Brine area	Pond Civil.	145.7	-
	Equipment cost	27.4	-2.5[1]
	Installation cost	48.9	-
	Indirect cost	64.2	-
	Contingency	71.3	-
	Brine area total	357.5	-2.5
Refinery factory	Equipment cost	34.4	-9.5[2]
	Installation cost	54.7	-
	Indirect cost	29.3	-
	Contingency	29.6	-
	Refinery factory total	148	-9.5
Total		505.5	-12

*Process capacity Li_2CO_3 25,000 ton/year

[1]Mg refinery: 30% reduction of reactor and liquid-solid separator (-2.5 million USD)

[2]Mg refinery: 30% reduction of reactor and liquid-solid separator (-3.7 million USD), 30% reduction from IX (-5.8 million USD)

19.4% cost reduction from total equipment cost.

No consideration of pond civil., installation cost, indirect cost and contingency.

Contingency = (Direct cost + Indirect cost) × 25%

Supplementary Table S16. CAPEX breakdown of commercial hard rock process and cost reduction less purifying Mg element.

Hard Rock Process

CAPEX*		Cost (M$)	Cost reduction (M$)
Equipment cost	Pyro area	46.2	-
	Hydro area	76.9	-9.5[1]
	Reagents & Utility	10.5	-
Total equipment cost		133.6	-9.5
Bulk and installation cost	Civil	13.4	-
	Concrete	20	-
	Structural steel	20	-
	Mechanical installation	13.4	-
	Piping	33.4	-
	Electrical & instrumentation	53.5	-
Total bulk and installation cost		153.7	-
Total direct cost		287.3	-9.5
Total indirect cost		94.4	-
Contingency		95.4	-
Total		477.2	-9.5

*Process capacity Li_2CO_3 25,000 ton/year

[1]Mg refinery: 30% reduction of reactor and liquid-solid separator (-3.7 million USD), 30% reduction from IX (-5.8 million USD).

7.3% cost reduction from total equipment cost.

No consideration of bulk and installation cost, indirect cost and contingency.

Contingency = (Direct cost + Indirect cost) × 25%

Supplementary Table S17. The amount of reagents usage and by-products generation. Assuming the concentration of each element after concentrating; Li, Mg, and Ca are 3.44, 7.96, and 0.14 g L^{-1}, respectively. The reagent $Ca(OH)_2$ are converted into 15.84 ton $(LC\ ton)^{-1}$ of $Mg(OH)_2$ cake assuming the moisture content of 30% in cake while only 0.98 ton $(LC\ ton)^{-1}$ of $Ca(CO)_3$ are generated at Ca refinery based on our P/P test.

	Compound	t/LC ton
**Reagents**	85% $Ca(OH)_2$	3.72
	99% Na_2CO_3	0.60
**By-products***	Mg cake; $Mg(OH)_2$	15.84
	Ca cake; $CaCO_3$	0.98

*assuming moisture content of 30%

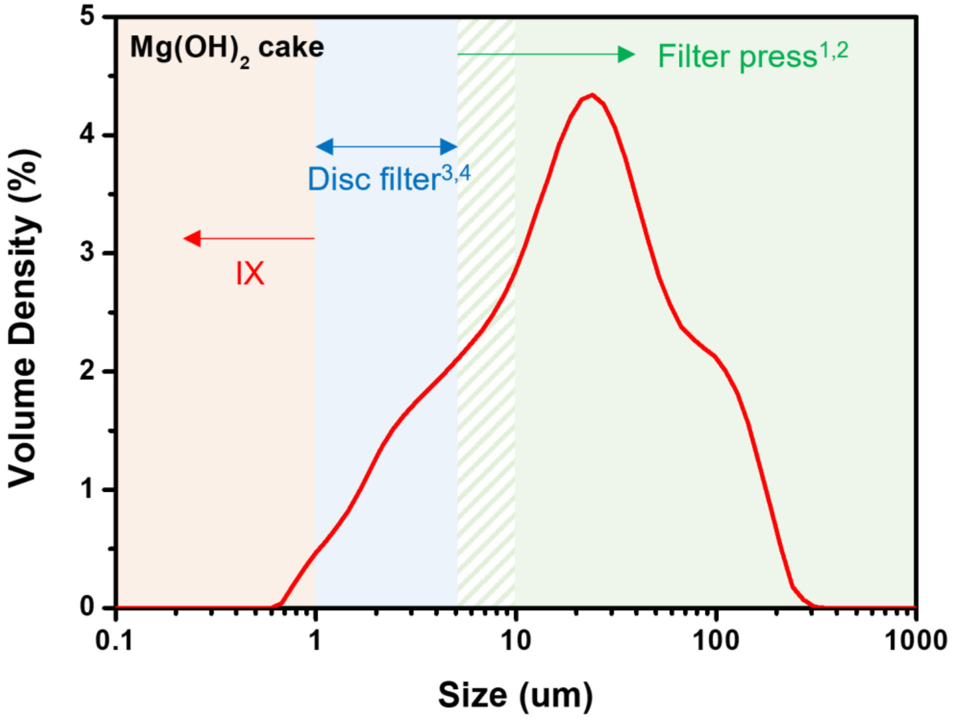

Supplementary Fig. S38. Particle size analyze result of $Mg(OH)_2$ cake; D10: 3.18, D50: 21.2, D90: 100. [1,2]Taeyoung (Korea), Ishigaki (Japan): Filter press; lowest filtration limit of 5 μm and guarantee >10 μm; 150~200K USD, [3,4]MKR (Germany), Alfalaval (Korea): Disc filter; guarantee 1~5 μm; 370~400K USD. The comparison of equipment cost was conducted based on same throughput. Additional disc filter and IX process are required to achieve *battery-grade* quality product which leads to considerable CAPEX and OPEX increasement.

Supplementary Table S18. OPEX breakdown of commercial brine process and cost reduction less purifying Mg element.

Brine Process

OPEX*		Cost ($/ton LCE)	Cost reduction ($/ton LCE)
Brine area	Consumables	66	-
	Reagent	1,751	-79.9[1]
	Product logistics cost	63	-
	Labor expenses	212	-
	General management expenses	80	-
	Maintenance	45	-
	Utility	85	-17[2]
	Waste disposal	126	-
	Brine area total	2,428	-96.9
Refinery factory	Consumables	133	-25[3]
	Reagent	83	-7[4]
	Labor expenses	115	-
	General management expenses	140	-
	Maintenance	62	-
	Utility	212	-50[5]
	Waste disposal	47	-9.4[6]
	Refinery factory total	792	-91.4
Total		3,221	-188.3

*Process capacity Li_2CO_3 25,000 ton/year
[1]$Ca(OH)_2$ reduction
[2]Mg refinery utility reduction of 20%
[3]IX resin refill
[4]HCl consumption for IX regeneration reduction of 20%
[5]IX process utility
[6]S/L separation cake waste reduction of 20%
5.85% cost reduction from OPEX

Supplementary Table S19. OPEX breakdown of commercial hard rock process and cost reduction less purifying Mg element.

Hard Rock Process

OPEX*	Cost ($/ton LCE)	Cost reduction ($/ton LCE)
Spodumene	4,301	-
Labor expenses	276	-
Reagent	865	-79.9[1]
Consumables	114	-
Utility	610	-50[2]
Maintenance	140	-
General management expenses	107	-
Waste disposal	581	-116.2[3]
Total	6,995	-246.1

*Process capacity Li_2CO_3 25,000 ton/year

[1] $Ca(OH)_2$ reduction

[2] Mg refinery & IX utility reduction of 30%

[3] Mg sludge waste and IX wastewater reduction of 20%

3.52% cost reduction from OPEX

Supplementary Table S20. The composition of brine and brine concentrate for Mg elimination process. Actual brine composition of certain brine in Argentina.

($g L^{-1}$)	Li	S	Ca	Mg	B	K	Na
Brine	0.863	3.218	0.766	2.34	0.645	8.947	104.25
Brine concentrate	4.2	11.1	0.17	10.1	2.83	37.3	85.3

Supplementary Table S21. The composition of brine concentrates after pilot plant (P/P) scale Mg elimination process. More $Ca(OH)_2$ than equivalent is used in general. Here, 1.3 equivalent of $Ca(OH)_2$ was needed to purify Mg to *battery-grade* from P/P test. In case 0.3 g L^{-1} ("LCM") or 0.5 g L^{-1} of Mg impurity in 10 g L^{-1} of Li solution are adjustable to Li_2CO_3 production process, \$79.9 or \$115.4 are reduced per 1 ton of Li_2CO_3 production. This calculation based on the on-site cost of 85% purity $Ca(OH)_2$ (on-site: 233 USD t^{-1}, off-site: 220 USD t^{-1}). Reduction on Mg refinery is more effective because the Mg refinery reagent ($Ca(OH)_2$) contains Ca that gives additional burdens to next Ca refinery step as shown in **Fig. 1c**.

$Ca(OH)_2$ (eq)	Li	S	Ca	Mg	B	K	Na
1.0	4.512	0.954	3.982	0.025	1.125	40.52	91.25
1.1	4.507	0.967	4.015	0.015	1.045	40.78	91.05
1.2	4.498	0.996	4.014	<0.003	1.23	41.06	92.49
1.3	4.415	1.008	3.811	<0.003	1.152	40.68	91.8
1.4	4.378	1.02	3.688	<0.003	1.1	40.56	91.15

Supplementary Table S22. CO_2 footprint of conventional brine process and CO_2 reduction less purifying Mg element.

**Brine***	CO_2 (eq.ton/ton)	Subject	CO_2 (eq.ton/ton)	CO_2 reduction (eq.ton/ton)
Material input	4.01 (42.3%)	Brine	-	-
Material input	4.01 (42.3%)	$Ca(OH)_2$	1.13 (11.9%)	-0.339 (-3.570%)[1]
Material input	4.01 (42.3%)	Na_2CO_3	2.03 (21.4%)	-
Material input	4.01 (42.3%)	NaOH	0.01 (0.1%)	-
Material input	4.01 (42.3%)	H_2SO_4	0.02 (0.2%)	-
Material input	4.01 (42.3%)	HCl	0.82 (8.7%)	-0.01 (-0.106%)[2]
Utility	0.00 (0.0%)	Surface water	-	-
Energy	1.23 (13.0%)	Natural gas	0.43 (4.5%)	-
Energy	1.23 (13.0%)	Electricity	0.8 (8.4%)	-0.23 (-2.415%)[3]
Waste	4.23 (44.7%)	Waste landfill	0.92 (9.7%)	-0.276 (-2.910%)[4]
Waste	4.23 (44.7%)	Wastewater	0.001 (0.01%)	-
Waste	4.23 (44.7%)	Flue gas (LNG combustion)	2.55 (26.9%)	-
Waste	4.23 (44.7%)	Flue gas (process)	0.76 (8.0%)	-
Total	9.47 (100.0%)	-	9.47 (100.0%)	-0.855 (-9.001%)

* Brine (in South America) / Conventional process

[1] Mg elimination material reduction

[2] IX regeneration material reduction

[3] IX operation energy and supplementary material input reduction

[4] $Mg(OH)_2$ waste reduction

Supplementary Table S23. CO_2 footprint of conventional hard rock process and CO_2 reduction less purifying Mg element.

**Hard Rock***	CO_2 (eq.ton/ton)	Subject	CO_2 (eq.ton/ton)	CO_2 reduction (eq.ton/ton)
Material input	3.55 (47.6%)	Spodumene	-	-
		$Ca(OH)_2$	0.24 (3.2%)	-0.072 (-0.966%)[1]
		Na_2CO_3	0.09 (1.2%)	-
		H_2O_2	0.19 (2.5%)	-
		NaOH	2.50 (33.5%)	-
		H_2SO_4	0.52 (7.0%)	-
		HCl	0.01 (0.1%)	-0.002 (-0.027%)[2]
Utility	0.001 (0.01%)	Process water	0.001 (0.01%)	-
Energy	2.33 (31.3%)	Natural gas	0.24 (3.2%)	-
		Electricity	2.09 (28.0%)	-0.230 (-3.086%)[3]
Waste	1.58 (21.1%)	Waste landfill	0.07 (0.9%)	-0.021 (-0.282%)[4]
		Wastewater sludge landfill	0.001 (0.01%)	-
		Wastewater	0.001 (0.01%)	-
		Flue gas (LNG combustion)	1.07 (14.4%)	-
		Flue gas (process)	0.43 (5.8%)	-
Total	7.45 (100.0%)	-	7.45 (100%)	-0.325 (-4.361%)

* Spodumene Ore concentrator / Conventional process

[1] Mg elimination material reduction

[2] IX regeneration material reduction

[3] IX operation energy and supplementary material input reduction

[4] $Mg(OH)_2$ waste reduction

Supplementary References

1. Mendieta-George, D., Pérez-Garibay, R., Solís-Rodríguez, R. & Rendón-Ángeles, J.C. Advantages of pH and Temperature Control in the Carbonation Stage for Li2CO3 Production with Sulphated Liquors. *ChemistrySelect*, **6**, 9517-9526 (2021).
2. Han, B., Haq, R.A.U. & Louhi-Kultanen, M. Lithium carbonate precipitation by homogeneous and heterogeneous reactive crystallization. *Hydrometallurgy*, **195**, 105386 (2020).
3. Zhang, Y. *et al.* LiNi0.90Co0.07Mg0.03O2 cathode materials with Mg-concentration gradient for rechargeable lithium-ion batteries. *J. Mater. Chem. A*, **7**, 20958-20964 (2019).
4. Pouillerie, C. *et al.* Effect of magnesium substitution on the cycling behavior of lithium nickel cobalt oxide. *J. Power Sources*, **96**, 293-302 (2001).
5. Kasnatscheew, J. *et al.* The truth about the 1st cycle Coulombic efficiency of LiNi 1/3 Co 1/3 Mn 1/3 O 2 (NCM) cathodes. *Phys. Chem. Chem. Phys.*, **18**, 3956-3965 (2016).
6. Xie, Q., Li, W. & Manthiram, A. A Mg-doped high-nickel layered oxide cathode enabling safer, high-energy-density Li-ion batteries. *Chem. Mater*. **31**, 938-946 (2019).
7. Zhang, M.-J. *et al.* Cationic ordering coupled to reconstruction of basic building units during synthesis of high-Ni layered oxides. *J. Am. Chem. Soc.* **140**, 12484-12492 (2018).
8. Dean, J., *Lange's handbook of chemistry*, 15th Ed., pp. 4.48 (1999).
